# The role of CYP-sEH derived lipid mediators in regulating mitochondrial biology and cellular senescence: implications for the aging heart

**DOI:** 10.3389/fphar.2024.1486717

**Published:** 2024-12-05

**Authors:** Ala Yousef, Liye Fang, Mobina Heidari, Joshua Kranrod, John M. Seubert

**Affiliations:** ^1^ Faculty of Pharmacy and Pharmaceutical Sciences, University of Alberta, Edmonton, AB, Canada; ^2^ Department of Pharmacology, Faculty of Medicine and Dentistry, University of Alberta, Edmonton, AB, Canada

**Keywords:** mitochondria, aging, PUFA, CYP, soluble epoxide hydrolase (sEH), epoxylipids

## Abstract

Cellular senescence is a condition characterized by stable, irreversible cell cycle arrest linked to the aging process. The accumulation of senescent cells in the cardiac muscle can contribute to various cardiovascular diseases (CVD). Telomere shortening, epigenetic modifications, DNA damage, mitochondrial dysfunction, and oxidative stress are known contributors to the onset of cellular senescence in the heart. The link between mitochondrial processes and cellular senescence contributed to the age-related decline in cardiac function. These include changes in mitochondrial functions and behaviours that arise from various factors, including impaired dynamics, dysregulated biogenesis, mitophagy, mitochondrial DNA (mtDNA), reduced respiratory capacity, and mitochondrial structural changes. Thus, regulation of mitochondrial biology has a role in cellular senescence and cardiac function in aging hearts. Targeting senescent cells may provide a novel therapeutic approach for treating and preventing CVD associated with aging. CYP epoxygenases metabolize N-3 and N-6 polyunsaturated fatty acids (PUFA) into epoxylipids that are readily hydrolyzed to diol products by soluble epoxide hydrolase (sEH). Increasing epoxylipids levels or inhibition of sEH has demonstrated protective effects in the aging heart. Evidence suggests they may play a role in cellular senescence by regulating mitochondria, thus reducing adverse effects of aging in the heart. In this review, we discuss how mitochondria induce cellular senescence and how epoxylipids affect the senescence process in the aged heart.

## 1 Introduction

Aging is a natural process involving progressive decline in biological systems, and decreased physiological reserve to handle stress leading to age-related disorders (Khan et al., 2017). Contemporary scientific understanding recognizes that the biological aging of an organ with respect to its structural and functional condition is heavily influenced by internal and external environmental factors (Moskalev, 2019). The pathophysiology of aging is often characterized by the dysregulation of interconnected crucial cellular functions, known as the hallmarks of aging, which include genomic instability, stem cell exhaustion, chronic inflammation, mitochondrial dysfunction, and cellular senescence (Lopez-Otin et al., 2013; Lopez-Otin et al., 2023). Cellular senescence is typically defined as a state where cells permanently stop dividing and lose their ability to proliferate. Senescent cells can secrete a significant number of inflammatory cytokines to the neighbouring cells, which contributes to a series of inflammatory responses. There are several types of cellular senescence, each are associated with different triggers and conditions. For example, replicative senescence which occurs during biological aging is characterized by telomere shortening, while stress-induced premature senescence (SIPS) is a telomere-independent process resulting from DNA damage caused by internal or environmental stress factors (Chang and Harley, 1995; Di Micco et al., 2021; Hu et al., 2022). The main factors contributing to cellular senescence include oxidative stress, DNA mutations, and mitochondrial mediated events. While mitochondria can activate senescence pathways and halt the cell cycle, our understanding of the biology is limited (Kumari and Jat, 2021). Therefore, understanding these connections could open new avenues for reducing the accumulation of pro-inflammatory senescent cells and preserving organ function.

**FIGURE 1:**
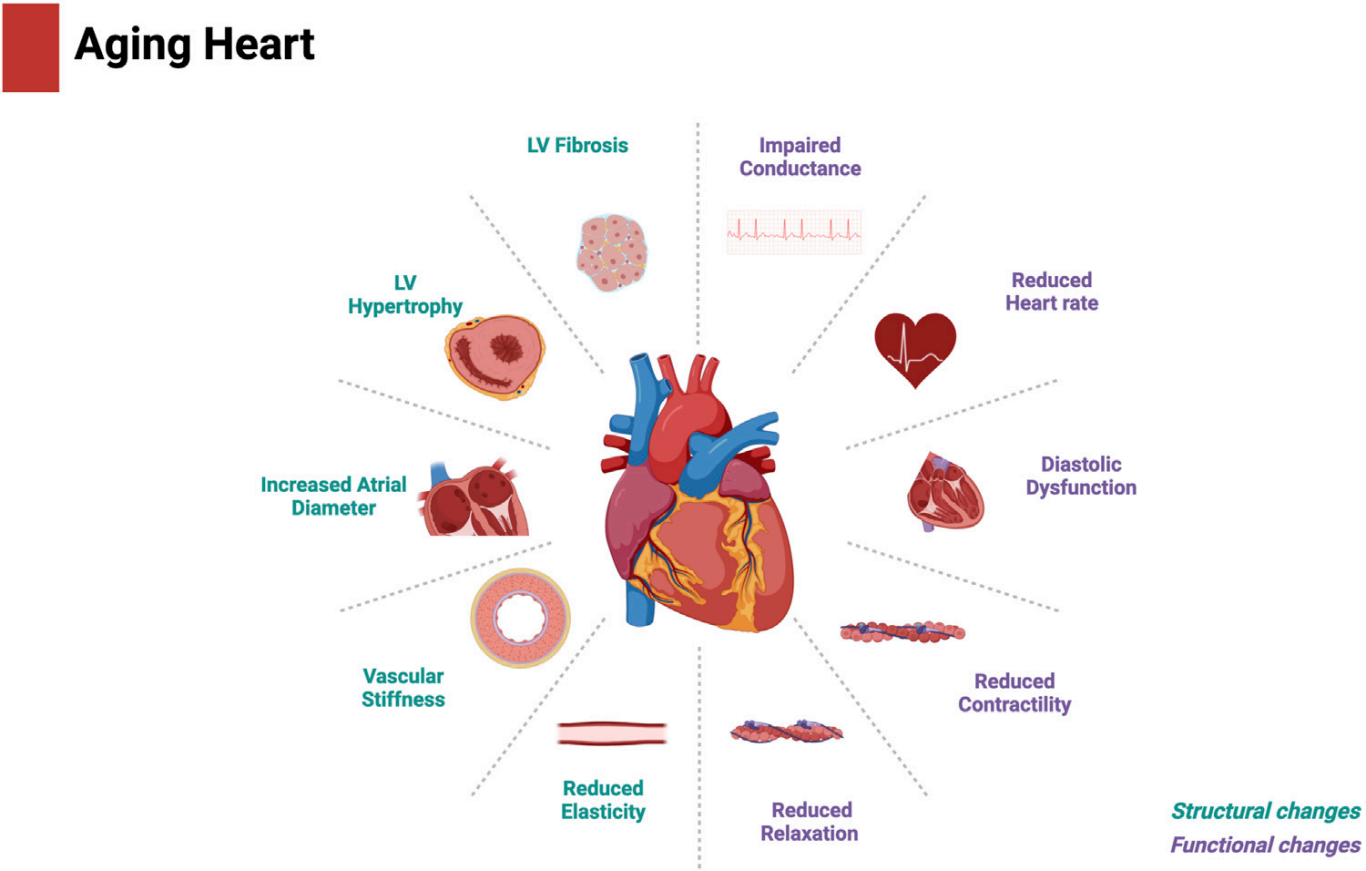
Structural and functional changes in the aged heart. Created with BioRender.com.

As individuals age, the heart undergoes structural and functional changes that significantly increase its susceptibility to stress. Aging is associated with several changes that impair myocardial contractile function and subsequently prevent the heart from meeting bodily circulatory demands. Age-associated cardiac impairment can often be attributed to pathogenic structural and biochemical changes including cardiomyocyte hypertrophy, chronic inflammation, and increased fibrosis (Cheng et al., 2009; Lakatta, 2015; Horn and Trafford, 2016). Together, these cellular and structural changes affect the heart at the organ level, leading to myocardial remodeling characterized by increased left ventricular mass, thickening and stiffening of the left ventricular walls and interventricular septum, and a decrease in left ventricular relaxation and diastolic function. With age, the heart becomes unable to respond to periods of increased cardiac demand because of a decline in contractility, cardiac output, and ejection fraction (Triposkiadis et al., 2019a; Triposkiadis et al., 2019b). Collectively, these changes increase the heart’s susceptibility to injury (Lakatta and Levy, 2003) (Figure 1). Cytochrome P450 (CYP)-derived metabolites of polyunsaturated fatty acids (PUFAs) has been demonstrated to regulate the progression of biological aging (Keshavarz-Bahaghighat et al., 2020). In this review, we discuss the role of CYP-derived epoxylipids in regulating mitochondrial biological processes and cellular senescence in the aged heart. We hypothesize that epoxylipids work to protect the aged heart against cellular senescence by regulating some aspects of the multifaceted biology of mitochondria.

## 2 CYP-sEH metabolism of polyunsaturated fatty acids (PUFAs)

**FIGURE 2:**
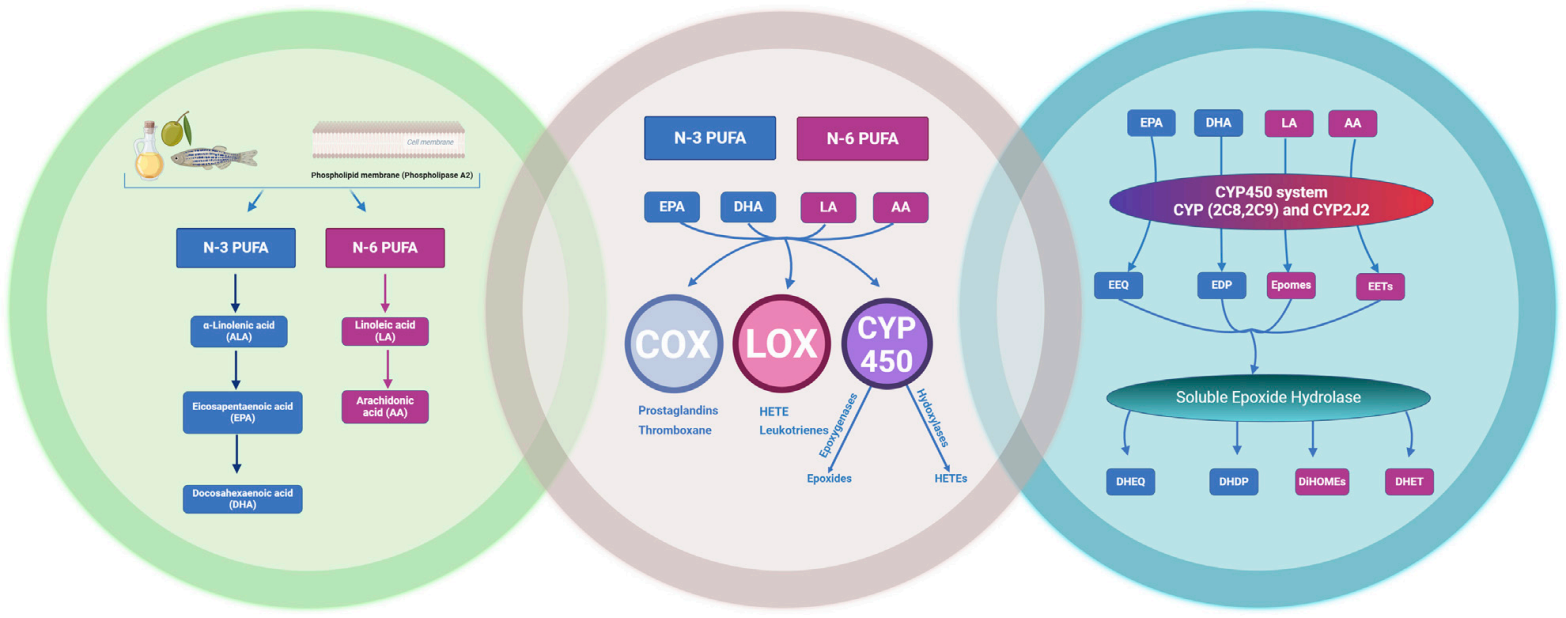
The metabolism of N-3 and N-6 polyunsaturated fatty acids (PUFAs). Eicosapentaenoic acid (EPA), docosahexaenoic acid (DHA), linoleic acid (LA) and arachidonic acid (AA) through the cyclooxygenases (COX), lipoxygenase (LOX), and cytochrome P450 (CYP450) pathways to their metabolites. The CYP450 system (CYP2C8, 2C9) and CYP2J2 produces bioactive epoxylipids epoxyeicosatetraenoic acids (EEQs), epoxydocosapentaenoic acids (EDPs), epoxyoctadecenoic acids (EpOMEs) and epoxyeicosatrienoic acids (EETs) which can be readily hydrolysed by soluble epoxide hydrolase into their respective diol metabolites. Dihydroxyeicosatetraenoic acid (DHEQ), dihydroxydocosapentaenoic acid (DHDP), dihydroxyoctadecenoic acid (DiHOmes) and dihydroxyeicosatrienoic acid (DHET). Created with BioRender.com.

PUFAs are essential fatty acids obtained from dietary sources such as fish, leafy greens, or supplements, and are characterized by possessing carbon-carbon double bonds at the N-3 or N-6 position (Gurr et al., 2002; Sokoła-Wysoczańska et al., 2018). Alpha linolenic acid (ALA 18:3), which is the primary source of N-3 PUFAs, undergoes a sequence of desaturation and elongation reactions, resulting in the formation of eicosapentaenoic acid (EPA 20:5) (Ander et al., 2003). EPA subsequently undergoes elongation, desaturation and β-oxidation reactions to form docosahexanoic acid (DHA 22:6). N-6 PUFAs including linoleic acid (LA 18:2) undergo metabolic transformation to form N-6 arachidonic acid (AA 20:4) through a similar series of desaturation and elongation reactions (Cunnane, 2003). Both N-3 and N-6 PUFAs are also subject to oxidative transformations through three main pathways including cyclooxygenases (COX), lipoxygenases (LOX) or CYP450 systems leading to the formation of numerous bioactive metabolites (Smith and Murphy, 2016) (Figure 2).

Different members of the CYP superfamily, such as CYP2C and CYP2J, are capable of metabolizing N-3 and N-6 PUFAs into short lived bioactive lipid mediators, exerting beneficial effects in various organs, particularly the cardiovascular system (Jamieson et al., 2017a). CYP2J and CYP2C isozymes are epoxygenases that metabolize N-3 PUFA eicosapentaenoic acid (EPA) and docosahexaenoic acid (DHA) into epoxyeicosatetraenoic acids (EEQ) and epoxydocosapentaenoic acids (EDP) respectively, and N-6 PUFA (LA and AA) into epoxyoctadecenoic acids (EPOMEs) and epoxyeicosatrienoic acids (EET) (Konkel and Schunck, 2011). Importantly, CYP-derived epoxides have a short half-life as a result of rapid metabolism primarily by soluble epoxide hydrolase (sEH) and, to a lesser extent, microsomal epoxide (mEH), turning them into less bioactive diol metabolites such as, dihydroxyeicosatetraenoic acid (DHEQ), dihydroxydocosapentaenoic acid (DHDP), dihydroxyoctadecenoic acid (DiHOME) and dihydroxyeicosatrienoic acid (DHET) (Jamieson et al., 2017a). Our understanding of the role the CYP-sEH axis of N-3 and N-6 PUFA metabolism has in the cardiovascular system is limited but is rapidly growing.

Published reports indicating changes in CYP-sEH mediated metabolism of PUFA correlate to increased CVD and decreased cardiac function (Bellien and Joannides, 2013; Doleželová et al., 2016; Caligiuri et al., 2017b). For example, left ventricular tissues obtained from dilated cardiomyopathy patients revealed a correlation between elevated expression of CYP-epoxygenases and epoxide hydrolases with altered PUFA metabolite profiles in comparison to non-failing control hearts (Sosnowski et al., 2022a). Further data obtained from left ventricular tissue of patients with ischemic cardiomyopathy demonstrated increased expression of sEH compared to age-matched non-failing controls and correlated with altered PUFA profiles (Jamieson et al., 2021). In another small clinical study investigating PAD patients, the relationship between plasma fatty acids and the prevalence of cardiovascular/cerebrovascular events indicated altered plasma oxylipins, oxygenated forms of PUFA, increased the odds of acute coronary events (Caligiuri et al., 2017a). Supported by Theken et al. findings of dysregulated CYP epoxygenase and sEH mediated metabolism in aged patients correlated with stable coronary artery diseases and comorbid obesity (Theken et al., 2012). Like human data, many studies showed positive correlation of CYP-sEH dysregulation in animal models with CVD (Aliwarga et al., 2018). For example, increased sEH expression is associated with adverse cardiac outcomes, including hypertension, left ventricular hypertrophy, and increased risk for heart failure progression (Monti et al., 2008). Additionally, a decline in CYP expression and a decrease in epoxylipids in the left ventricle of aged female rats and a decrease in epoxylipid levels, like EETs, in aged hypertensive male rats have been reported, leading to endothelial dysfunction and age-related kidney diseases (Doleželová et al., 2016). Together these reports highlight the correlation between altered CYP-sEH metabolism and cardiac dysfunction in diseased human and animal models.

While alteration in CYP-sEH metabolism have been observed in many CVD, the implications of increasing CYP and epoxylipids and/or inhibiting sEH in protecting against CVD have been extensively reviewed elsewhere (Westphal et al., 2015; Schunck et al., 2018; Valencia et al., 2022). For example, mice with cardiac overexpression of CYP2J2 exhibited enhanced cardiac function, reduced myocardial hypertrophy, and decreased fibrosis, resulting in the improvement of overall structure and function of the heart (Zhang et al., 2009; Liu et al., 2011; Westphal et al., 2013; He et al., 2015). Additionally, cardiomyocytes from mice with cardiac overexpression of CYP2J2 demonstrated decreased levels of remodeling proteins, such as collagen type I and transforming growth factor β (TGF-β), in response to Angiotensin II compared to their wild-type counterparts (He et al., 2015). When these CYP2J2 transgenic mice were subjected to pressure-overload or prolonged infusion of isoproterenol, they exhibited diminished hypertrophy and fewer arrhythmogenic events (Westphal et al., 2013).

N-3 and N-6 derived epoxylipids including EETs, EEQs, and EDPs play significant roles in cellular signaling and metabolism, often marked by cardioprotective effects (covered extensively, elsewhere (Imig et al., 2022)), which has led to research developing novel stable synthetic analogs mimicking their properties (Sudhahar et al., 2010; Campbell et al., 2017). For example, a 11,12-EET analog, (S)-2-(11-(nonyloxy) undec-8(Z)-enamido) succinic acid (NUDSA), resulted in improved left ventricular function, reduced myocardial fibrosis, and remodeling following MI (Cao et al., 2017). Similarly, the oral administration of a 14,15-EET analog, EET-B, to hypertensive rats subjected to MI-induced heart failure resulted in a decrease in mortality, improved cardiac function, and decreased inflammation and macrophage infiltration (Neckar et al., 2019). Another analog, UA-8 (13-(3-propylureido) tridec-8-enoic acid), a synthetic dual-action compound with EET-mimetic and sEH-inhibitory, also increased isolated hearts’ resistance to cardiac ischemia (Batchu et al., 2011). The 14,15-EET analog, EET-A, decreased cardiac hypertrophy and reduced ventricular arrhythmias following MI in hypertensive rats (Cervenka et al., 2018). Moreover, EET-A improved survival and reduced cardiac hypertrophy in hypertensive rats with congestive heart failure (Kala et al., 2021). Recent evidence has demonstrated cardioprotective and anti-inflammatory properties of a SA-22, a molecule developed to mimic the structure and function of a CYP-derived metabolite N-3 PUFA, 19,20-EDP, which preserved mitochondrial function (Kranrod et al., 2024). These findings emphasize the potential of using novel synthetic analogs as therapeutic agents toward cardiovascular injury.

Extensive research has investigated the potential therapeutic benefit of inhibiting sEH in various cardiovascular injury models such as ischemic heart diseases, arrythmias, hypertrophy and heart failure (Imig and Hammock, 2009; Oni-Orisan et al., 2014; Schunck et al., 2018). Data from rodent and canine models clearly demonstrate a reduced level of ischemia injury following administration of sEH inhibitors (Gross et al., 2008; Chaudhary et al., 2010; Akhnokh et al., 2016; Islam et al., 2017; Darwesh et al., 2019b; Stevenson et al., 2019). An important mechanism involves preserving mitochondria, which limits cardiac dysfunction. In mice subjected to permanent coronary artery occlusion, treatment with *cis*-4-[4-(3-adamantan-1-yl-ureido) cyclohexyloxy] benzoic acid (*c*AUCB) led to significant increase in the biologically active epoxylipids and significantly reduced infarct size in myocardial ischemia (Neckář et al., 2012). Interestingly, the protective effect demonstrated was abolished when administering selective EET antagonists, suggesting epoxylipids have a primary role in *c*AUCB-mediated cardioprotection (Neckář et al., 2012). Furthermore, inhibition of sEH protected against electrical conductance abnormalities and heart arrythmias (Monti et al., 2008; Neckář et al., 2012; Sirish et al., 2013). In addition, administration of sEH inhibitors to mice with thoracic aortic constriction resulted in reduced cardiac remodeling and electrical abnormalities which reduced arrhythmias in mice (Sirish et al., 2016). In rodent models of cardiac hypertrophy, treatment with sEH inhibitors decreased atrial and ventricular arrhythmias and effectively reduced isoproterenol-induced cardiac hypertrophy (Monti et al., 2008; Sirish et al., 2013). Likewise, administration of an sEH inhibitor to mice subjected to acute left anterior descending (LAD) artery occlusion had reduced cardiac fibrosis and improved left ventricular function (Kompa et al., 2013). Additionally, treatment with sEH inhibitors have been shown to improve heart function, reducing cardiac hypertrophy and fibrosis in heart failure models (Qiu et al., 2011; Merabet et al., 2012; Stevenson et al., 2019). The combination of sEH inhibitors and epoxylipids has been evaluated, where co-treatment reduced ventricular fibrillation and cardiac hypertrophy (Cervenka et al., 2018) and amplified the cardioprotective effect in left ventricular function following MI (Hrdlička et al., 2019). Mechanisms responsible for reducing cardiac fibrosis and hypertrophy include improved mitochondrial function, reduced oxidative stress and inflammation (Qiu et al., 2011; Jamieson et al., 2017a; Imig et al., 2022). Inhibition of sEH resulted in reduced cardiac injury caused by systemic inflammation following exposure to lipopolysaccharide (LPS) (Samokhvalov et al., 2018; Yousef et al., 2024). Further data demonstrated the cardiomyocyte targeted knockdown of sEH was enough to preserve cardiac function and limit inflammation following LPS exposure (Sosnowski et al., 2022b). Together, these data suggest the cardioprotective effects of inhibiting sEH potentially involves attenuating an exaggerated inflammatory response (Samokhvalov et al., 2018; Sosnowski et al., 2022b; Yousef et al., 2024).

## 3 Cellular senescence

Cellular senescence is a state of stable cell cycle arrest where cells cease to proliferate but remain metabolically active, often exhibiting a pro-inflammatory secretory phenotype. Senescent cells were first discovered by Hayflick and Moorhead in 1961 following their observation in cultured human fibroblasts had a limited capacity for cell division and entered a stable, irreversible cell cycle arrest (Hayflick and Moorhead, 1961). The accumulation of senescent cells in aged organisms contributes to a decline in overall health and increases susceptibility to disease. Telomere shortening, epigenetic modifications, DNA damage, oxidative stress and mitochondrial mediated events are known to accelerate the onset of cellular senescence. Replicative senescence is correlated with the normal aging process as cells divide and replicate over time. Whereas SIPS, occurs independently of the chronological aging process, and can be triggered in young cells by a stress stimulus leading to DNA damage, oxidative stress and changes to mitochondrial activities, functions and behaviors (Balaban et al., 2005; Childs et al., 2015; Zhu et al., 2018). Senescent cells produce large amounts of immune modulators, inflammatory cytokines, growth factors, and chemokines which act in both a paracrine and endocrine manner known as the senescence-associated secretory phenotype (SASP) (Lopes-Paciencia et al., 2019). The induction of SASP has immediate impact on the surrounding cells but can also affect the whole organism. As individuals age, the number of senescent cells increases, and pro-inflammatory cytokines produced by SASP-positive cells contribute to chronic inflammation associated with aging (Khavinson et al., 2022).

Cellular senescence plays a significant role in cardiac aging, with senescent cells in the heart contributing to a decline in function, such as reduced contractility and impaired mitochondrial function or activities, like altered oxidative phosphorylation (OxPhos), calcium regulation or membrane potential (Anderson et al., 2019; Shimizu and Minamino, 2019). Studies have shown that promoting cellular senescence can accelerate the onset of age-related heart conditions. For example, senescence-accelerated mice on a high-fat, high-salt diet showed an increase in senescent endothelial cells in the heart, correlating with diastolic dysfunction and left ventricular hypertrophy (Gevaert et al., 2017). Anderson et al. demonstrated that, with aging, both human and murine cardiomyocytes displayed a senescent-like phenotype, including the secretion of atypical SASP factors like endothelin 3 (Edn3), Tgfβ2 and growth differentiation factor-15 (GDF15) (Anderson et al., 2019). Moreover, conditioned culture medium from aged cardiomyocytes induced fibroblast activation and senescence, suggesting an interaction between senescent cardiomyocytes and fibroblasts during cardiac aging and dysfunction (Anderson et al., 2019). These findings indicate senescent cells can actively impair the function of surrounding cells. The accumulation of senescent cardiomyocytes, leads to a functional decline, characterized by decreased contractility, increased cell size, and changes to mitochondrial function and activity, ultimately compromising cardiac performance. As these senescent cells accumulate, they disrupt intercellular communication, exacerbate chronic inflammation, and contribute to cell death, culminating in cardiac dysfunction (Tang et al., 2020).

### 3.1 Markers of cellular senescence

**FIGURE 3:**
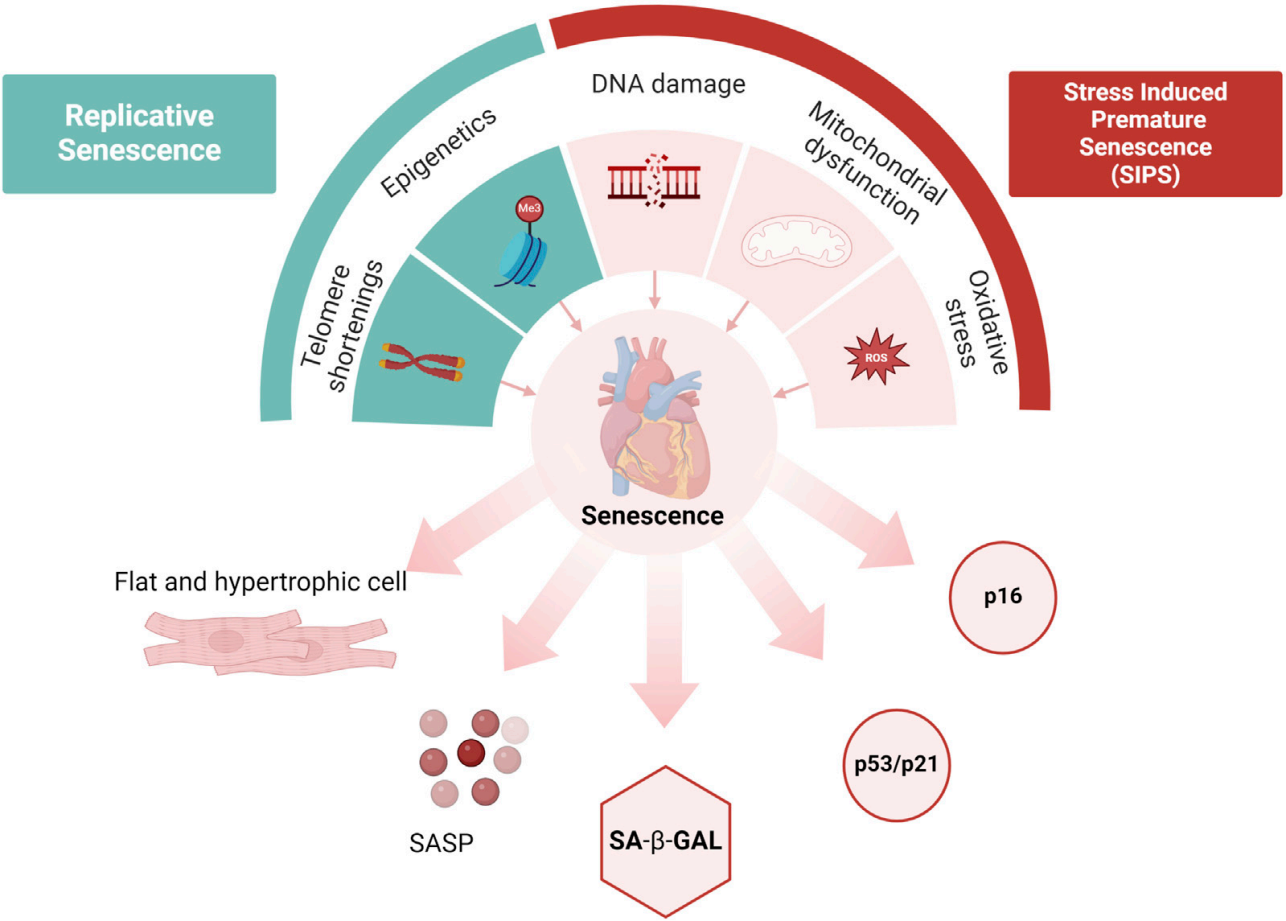
Cellular senescence. Replicative senescence is associated with the aging process, while stress-induced senescence (SIPS) can be prematurely triggered by acute stressors. Causes of senescence include epigenetic changes, telomere shortening, DNA damage, mitochondrial dysfunction, and oxidative stress. Hallmarks of senescence include structural changes and pathway activations (such as p53/p21 and p16), accumulation of SA-β-gal, and the release of Senescence-Associated Secretory Phenotype (SASP). Created with BioRender.com (Ahmad et al., 2015).

The absence of a reliable marker for detecting senescent cells, especially *in vivo*, has historically been a significant challenge in the field of senescence research. Therefore, various markers have been utilized to identify these cells. These include the presence of β-galactosidase (β-gal) activity, the expression of tumor suppressors, cell cycle inhibitors such as p53/p21 and p16, DNA damage markers and telomere shortening (Figure 3). Cellular senescence is typically characterized by increased β-gal activity, which is linked to active autophagy and high lysosomal content. β-gal is a lysosomal hydrolase that promotes the breakdown of many β-d-galactoside substrates including lactose, keratin sulfates and sphingolipids within the lysosomal environment (Krishna et al., 1999). Increased activity of lysosomal β-gal is associated with increased cellular senescence, marked by an increase in both the number and size of lysosomes (Robbins et al., 1970; Kurz et al., 2000; Gary and Kindell, 2005). Upregulated β-gal activity has been observed in the organs of elderly humans and other animals, indicating that cellular senescence is a characteristic of biological aging (Dimri et al., 1995; Judge and Leeuwenburgh, 2007). Distinguishing between senescent and non-senescent cells either in *in vivo* or *in vitro* models is challenging, however, co-immunoprecipitation of β-gal with specific cell markers such as troponin T or α actinin for cardiomyocyte, α-smooth muscle actin (α-SMA) for myofibroblast and vimentin for fibroblast provides a way to identify the senescent cell types such as cardiomyocytes, myofibroblasts and fibroblasts (Meyer et al., 2016). Anderson et al. (2019), found that β-gal activity was increased in aged mice, particularly in cardiomyocytes when they are co-labelled with troponin C (Anderson et al., 2019).

Tumor suppressor p53 serves as an important marker in cellular senescence, which is often associated with excessive cellular stress such as DNA damage (Mijit et al., 2020). The activity of p53 is modulated by post-translational modifications such as ubiquitination, phosphorylation, and acetylation, allowing it to positively regulate genes involved in cell cycle arrest and senescence (Bode and Dong, 2004). Activation of the p53 pathway happens through a series of kinase cascades involving ATM, ATR, and CHK1/CHK2 making it a key transcription factor in determining cell fate (Shi et al., 2021). Recent research has highlighted the role of p53-dependent senescence in the cardiovascular system. In aged cardiac endothelial cells, reduced SIRT1 expression leads to increased p53 acetylation, leading to senescence and endothelial dysfunction (Ota et al., 2007). Elevated p53 levels are found in patients with end-stage heart failure and cardiomyopathy, suggesting a role in heart dysfunction (Song et al., 1999; Birks et al., 2008). While deletion or inhibition of p53 prevented senescence in many cardiac cell types such as cardiomyocyte, fibroblasts, endothelial cells and vascular smooth muscles (Zhu et al., 2013; Gu et al., 2018; Yokoyama et al., 2019).

One of the downstream targets of p53 is p21 (*CDKN1A*), a member of the cyclin-dependent kinase inhibitors (CDKI) and essential component for p53-mediated cell cycle arrest at the G1/S or G2/M checkpoint (Yosef et al., 2017). Studies have shown that the expression of p21 was found to be increased in both murine and human senescent cells, leading to cell cycle arrest specifically at the G1 phase. The upregulation of p21 expression has been extensively examined as a potential biomarker for senescence in response to various stressors (Jia et al., 2017; Sawaki et al., 2018; Lee et al., 2020; Cai et al., 2021). These results suggest that cellular senescence is associated with p53 and p21 activation and can be used as a biomarker of senescence in the heart.

Another significant pathway involved in cellular senescence is the p16/Rb pathway. Phosphorylation of the retinoblastoma protein (Rb) by cyclin-dependent kinase 4 (CDK4) is a crucial step in cell cycle division. Increased expression of p16INK4a is observed in nearly 50% of ventricular myocytes in the aged and diseased heart (Chimenti et al., 2003). Consequently, the accumulation of these p16-positive cells is believed to have a negative impact on longevity, suggesting a potential association between cellular senescence marked by p16 and the aging process (Baker et al., 2016). In engineered mouse models, such as p16-3MR, enabling the selective elimination of p16-positive senescent cells, the clearance of these cells has shown promising results in delaying age-related disorders across multiple tissues and organs, including the heart (Baker et al., 2016).

During prenatal and early neonatal development, telomere shortening is extensive, where the occurrence of cardiomyocyte proliferation observed during the first (Porrello et al., 2011) and third weeks of life (Naqvi et al., 2014). However, in postnatal life, telomere shortening proceeds at a significantly slower rate, losing only 13 base pairs per year in the left ventricle, compared to other tissues such as the kidneys, which demonstrate an annual reduction of 30–60 base pairs (Takubo et al., 2002). The primary mechanism underlying telomere-driven aging is based on the concept that continuous cellular turnover leads to a gradual depletion of telomere length, ultimately resulting in short and dysfunctional telomeres (Moslehi et al., 2012; Sharifi-Sanjani et al., 2017). Evidence from human arterial endothelium has demonstrated that telomere shortening is age-dependent and accelerated by CVD risk factors (Voghel et al., 2007). However, several studies state that cellular senescence extends beyond telomere shortening. For example, post-mitotic cardiomyocyte senescence is triggered by telomere damage independent of telomere length (Anderson et al., 2019). Notably, telomere specific DNA damage promoted cellular senescence in cardiomyocytes (Anderson et al., 2019), which was consistent with data demonstrating DNA damage repair promoted both cellular senescence and cardiac aging (Lyu et al., 2018). DNA damage induced by bacterial toxicity can subsequently trigger cellular senescence (Blazkova et al., 2010; Wei et al., 2022; Yousef et al., 2024). Similarly, oxidative DNA damage characterized by the accumulation of 8-hydroxy 2 deoxyguanosine has been observed in cardiotoxic models (Topcu et al., 2022; Yousef et al., 2024). Altogether, these studies report telomere and DNA damage are critical drivers and markers of cardiomyocyte senescence.

Cellular senescence is commonly associated with changes in cellular morphology. One of the key features of dysfunctional cardiomyocytes in aged myocardial tissues is pathological hypertrophic growth, which is commonly associated with senescence (Cui et al., 2018). Senescent cells demonstrate enlarged and flattened shape with vacuolization of the cell body (Ball and Levine, 2005). However, these morphological changes have been seen in three different cell types in the heart: vascular smooth muscle cells (VSMC), cardiomyocytes and endothelial cells (Ball and Levine, 2005; Ota et al., 2007; Huang et al., 2021). Moreover, increased expression of hypertrophic genes such as Myh7 and Acta1 was reported in cardiomyocyte senescent cells (Anderson et al., 2019).

### 3.2 Senescence-associated secretory phenotype

The senescence-associated secretory phenotype (SASP) describes the primary characteristics and relevant biomarkers released by senescent cells, this includes the elevated production of pro-inflammatory immune modulators, cytokines, growth factors, chemokines, and extracellular matrix proteases. While these factors influence the immediate environment of a SASP-positive cell, they also function as remote endocrine signals. As a result, large-scale induction of the SASP significantly contributes to systemic aging. Senescent cells utilize the SASP to communicate both internally and with their surrounding microenvironment (Tang et al., 2020).

The onset of cellular senescence is marked by an increase in the expression of pro-inflammatory interleukins (IL-6, IL-8, IL-11, IL-1α and IL1β), monocyte chemoattractant protein (MCP-1), tumor necrosis factor (TNF-α), TGFβ, vascular endothelial growth factor (VEGF), insulin-like growth factor, chemokine CXC motif ligands 1 and 2 (CXCL1, CXCL2), GDF-15 and Edn3 (Coppe et al., 2008; Anderson et al., 2019; Kirschner et al., 2020). The SASP also includes a plethora of other inflammatory cascades, for example, during senescence increased both IL-1α and IL-1β proteins activate NF-kB-mediated protein transcription, a major feed-forward mechanism in the SASP (Lau et al., 2019). Aging and age-related diseases can be attributed to the accumulation of senescent cells in various tissues (Childs et al., 2015). Due to the SASP, senescent cells influence nearby cells and reinforce their own senescent state via an autocrine loop (Young and Narita, 2009). Examples include IL-6 binding to its receptor or IL-8 interacting with the CXCR2 receptor thereby reinforcing the inflammatory state of senescent cells (Kuilman et al., 2008). Thus, it has been hypothesized that eliminating senescent cells could slow down biological aging (Amaya-Montoya et al., 2020; Pignolo et al., 2020). For example, IL-11, a pro-inflammatory SASP cytokine, contributes to the induction of senescence in cardiac fibroblasts. Inhibition of IL-11 has been shown to reduce senescence biomarkers and inflammation, thereby promoting healthy aging and extending lifespan (Widjaja et al., 2024). Additionally, senolytic drugs which can be used to selectively remove senescent cells, delaying the aging process (Yosef et al., 2016). This suggest that inhibiting or eliminating senescent cells, or suppressing the SASP, could potentially reverse aging.

Recent research indicates the senescent phenotype, including the SASP, is not a uniform or static entity but rather a complex and evolving network that varies significantly based on cell type, triggering factors, and the stage of senescence (Sharpless and Sherr, 2015; Hernandez-Segura et al., 2017). This differential expression pattern is a key factor contributing to the observed heterogeneity in the senescence program (Sharpless and Sherr, 2015). Highlighted in a study utilizing transcriptomic approaches demonstrating SASP genes differ substantially across various time points and cell types (Hernandez-Segura et al., 2017). Moreover, various cardiac cell types undergo senescence, influencing both aging and CVD (Tang et al., 2020; Hu et al., 2022). These including cardiomyocytes, cardiac fibroblasts, cardiac endothelial cells, vascular smooth muscle cells and cardiac stem cells Table 1.

**TABLE 1:** Cellular senescence in cardiac cell types.

Cell type	Model	*In vitro*/*In vivo*	Cell Marker(s)	Disease/Stressor(s)	Aging/Senescence Marker(s)	SASP Component(s)	Pathological Effect(s)	References
Cardiomyocytes	Doxorubicin induced cardiotoxicity	Both	Wistar rat, neonatal rat cardiomyocytes, H9c2	Doxorubicin	mtDNA damage, p16, SA-β-Gal		Cardiac dysfunction and accelerated aging	Mitry et al. (2020)
Cardiomyocytes	Cardiomyocyte-specific model	Both	α-actinin, neonatal rat cardiomyocytes	Cardiac aging and IGF-1	SA-β-Gal, p21	IL-1α, IL-1β, IL-6	Cardiac hypertrophy and fibrosis	Ock et al. (2016)
Cardiomyocytes	Primary cardiomyocytes	*In vitro*	Neonatal rat cardiomyocytes	Hypoxia reoxygenation	SA-β-Gal, p16		Premature senescence	Zhang et al. (2007)
Cardiomyocytes	Primary cardiomyocytes	*In vivo*	Troponin C, α-actinin	Cardiac aging	DNA damage, SA-β-Gal, p53, p21, p16	GDF15, TGF-β, Edn3	Cardiac hypertrophy and fibrosis	Anderson et al. (2019)
Cardiomyocytes	Primary cardiomyocytes	*In vivo*	Primary cardiomyocytes isolated from aged mice	Cardiac aging	SA-β-Gal	TGF-β	Age-related cardiac dysfunction	Lyu et al. (2018)
Cardiomyocytes	Mouse hearts	*In vivo*	α-actinin	Diabetes and cardiac aging	p21, p53		Cardiac dysfunction	Kosugi et al. (2006)
Cardiomyocytes	H9c2 cells	*In vitro*		Nanoplastics	SA-β-Gal, p16, p21, DNA damage	IL-6, TNF-α, IL-1β	Cardiomyocyte Senescence	Wang et al. (2024)
Cardiomyocytes	H9c2 cells	*In vitro*		Doxorubicin	SA-β-Gal, p16 p21	IL-1β, IL-6, IL-12, TNF-α	Cardiomyocyte Senescence	Huang et al. (2021)
Cardiomyocytes	H9c2 cells	*In vitro*		Doxorubicin	SA-β-Gal, p16		Cardiomyocyte Senescence	Pillai et al. (2021)
Cardiomyocytes	Rap1 knockout mice	*In vivo*	α-actinin, primary cardiomyocytes isolated from aged mice	Cardiac aging	p53		Cardiac hypertrophy and dysfunction	Cai et al. (2021)
Cardiac endothelial cells	Porcine coronary artery	*In vitro*	Endothelial cells	High glucose	SA-β-Gal, p53, p21	VCAM-1	Endothelial cell aging	Khemais-Benkhiat et al. (2020)
Cardiac endothelial cells	Heart failure	*In vivo*	Endothelial specific knockout	Isoproterenol	p53, p21	ICAM-1	Heart failure	Katsuumi et al. (2018)
Cardiac endothelial cells	Coronary artery diseased patients	*In vitro*	Von Willebrand Factor, CD31	Serial passages	Telomere shortening, SA-β-Gal		Endothelial dysfunction and CVDs	Voghel et al. (2007)
Cardiac endothelial cells	Coronary artery diseased Patients	*In vitro*	Factor VIII	Atherosclerosis	SA-β-Gal	ICAM-1	Endothelial dysfunction and atherosclerosis	Minamino et al. (2002)
Endothelial cells	Human umbilical vein endothelial cells (HUVECs)	*In vitro*		High glucose	SA-β-Gal, telomerase activity		Endothelial senescence and atherosclerosis	Hayashi et al. (2006)
Cardiac endothelial cells	Senescence-accelerated mouse model	*In vivo*	CD31	Heart failure with preserved ejection fraction	p53, SA-β-Gal	ICAM-1	Endothelial senescence and heart failure	Gevaert et al. (2017)
Cardiac endothelial cells	Human aortic endothelial cells	*In vitro*		LDL exposure	SA-β-Gal, DNA damage, p53, p16		Vascular endothelial senescence and atherosclerosis	Wang et al. (2018)
Endothelial cells	Human umbilical vein endothelial cells	*In vitro*		MiR-34a and serial passages	SA-β-Gal		Endothelial cellular senescence	Ito et al. (2010)
Endothelial cells	Endothelial specific knockout mice	*In vivo*	Isolectin B4	STZ-induced diabetes and ischemia	p53, p21		Vascular endothelial senescence	Yokoyama et al. (2019)
Endothelial cells	Human umbilical vein endothelial cells (HUVECs)	*In vitro*		Sirt1 inhibition and hydrogen peroxide	SA-β-Gal, p53		Premature senescence and endothelial dysfunction	Ota et al. (2007)
Cardiac fibroblasts	MI	*In vivo*	Vimentin	MI	DNA damage, p21, SA-β-Gal		Cardiac fibrosis	Shibamoto et al. (2019)
Cardiac fibroblasts	Ventricles of neonatal rats, spontaneously hypertensive rats	Both	Fibroblast isolation, α-SMA	Hypertension induced fibrosis	p53, p21, SA-β-Gal	IL-6, IL-1β, TGF-β	Cardiac fibroblasts senescence and hypertrophy	Li et al. (2019)
Cardiac fibroblasts	Neonatal and adult mouse models	*In vivo*	PDGFRα	MI	p53, p16, SA-β-Gal	IL-1α, IL-6, CCl2, VEGF, MMP2	Fibroblast senescence and cardiac fibrosis	Feng et al. (2019)
Cardiac fibroblasts	Human heart biopsies and mouse model	*In vivo*	PDGFR-α, α-SMA	Transverse aortic constriction	p21, p16, SA-β-Gal		Cardiac hypertrophy and dysfunction	Meyer et al. (2016)
Cardiac fibroblasts	Mouse hearts	*In vitro*	Isolation of cardiac fibroblasts	Palmitate treatment	SA-β-Gal	TNF-α, IL-6, CXCL2, IL-1β, IL-18	Reduced contractile function	Sokolova et al. (2017)
Cardiac fibroblasts	Human biopsies	*In vitro*	Vimentin	Atrial fibrillation	P16, p21, SA-β-Gal		Premature senescence and cardiac fibrosis	Xie et al. (2017)
Cardiac fibroblasts	Mouse hearts	*In vivo*	α-SMA	Myocardial infarction	p53, p16, p21, SA-β-Gal	IL-6, CXCL1, CXCL2, MCP-1	Cardiac fibrosis	Zhu et al. (2013)
Cardiac fibroblasts	Mouse hearts	Both	Vimentin	miR-17-3p transgenic mouse model and hydrogen peroxide	SA-β-Gal		Cardiac aging	Du et al. (2015)
Cardiac fibroblasts	Mouse hearts	*In vivo*	Vimentin	MI	p53, SA-β-Gal		Heart injury	Sarig et al. (2019)
Cardiac fibroblasts	Mouse hearts	*In vivo*	Vimentin	Cardiac aging	p16, p53, p21, SA-β-Gal	TGF-β	Age-related cardiac fibrosis and dysfunction	Sawaki et al. (2018)
Cardiac fibroblasts	Primary mouse fibroblast	*In vitro*		Serial passages	SA-β-Gal		Age-related cardiac fibrosis	Wang et al. (2015b)
Cardiac fibroblasts	MI	*In vivo*	α-smooth muscle actin	MI	SA-β-Gal, p53, p21	VEGF	Accelerated heart failure post-MI	Jia et al. (2017)
Cardiac fibroblasts	Mouse hearts	*In vivo*	Vimentin	MI, cryoinjury	SA-β-Gal, p53, p21		Cardiac fibrosis	Zhang et al. (2024)
Vascular smooth muscle cells	VSMC specific knockout	*In vivo*	SM22α	Atherosclerosis and aging		IL-6, CCL2	Inflammation and atherosclerosis risk	Song et al. (2012)
Vascular smooth muscle cells	Human vascular smooth muscle cells	*In vitro*		IL-1β induced senescence	SA-β-Gal, p16, p21, telomerase activity	TNF-α, IL-6, MCP-1	Inflammation and atherosclerosis risk	Zhao et al. (2021)
Vascular smooth muscle cells	Mouse Aorta	*In vitro*		Angiotensin II, Aldosterone	SA-β-Gal, p16, p21, DNA damage	IL-6, TNF-α	Vascular remodeling and senescence	Gan et al. (2021)
Vascular smooth muscle cells	Human aortic smooth muscle cells	*In vitro*		hypertensive aged patients	p21, p16, SA-β-Gal		Vascular senescence and CVDs	Ma et al. (2022)
Vascular smooth muscle cells	Rat Aorta	*In vitro*	Isolation of VSMCs	Angiotensin II	SA-β-Gal, p53, p21, p16		Vascular senescence and CVDs	Min et al. (2007)
Vascular smooth muscle cells	Aortic mice plaque	*In vivo*		Ionizing radiation	p16, p21, DNA damage	MCP-1	Atherosclerosis	Yamamoto et al. (2021)
Vascular smooth muscle cells	Rat Aorta	*In vitro*	Isolation of VSMCs	Serial passages	SA-β-Gal, p53, p21		Vascular aging	Tan et al. (2019)
Vascular smooth muscle cells	Human aortic smooth muscle cells	*In vitro*		Serial passages	SA-β-Gal, DNA damage, p53, p21		Vascular senescence and CVDs	Chen et al. (2021)
Vascular smooth muscle cells	human atherosclerotic samples	*In vitro*	α-smooth muscle actin	Atherosclerosis	SA-β-Gal, p53, p16, p21	IL-1β, IL-6, IL-8, MCP-1	Vascular senescence and atherosclerosis	Minamino et al. (2003)
Vascular smooth muscle cells	Human aortic VSMC	*In vitro*		Angiotensin II	SA-β-Gal, p21, p16	IL-6	Vascular aging	Tsai et al. (2016)
Vascular smooth muscle cells	Human aortic VSMC	*In vitro*	α-smooth muscle actin	Bleomycin	SA-β-Gal, p16, p53, DNA damage	IL-1α, IL-6, IL-8, MCP-1, MMP9	Vascular senescence and atherosclerosis	Gardner et al. (2015)
Vascular smooth muscle cells	Human and mouse atherosclerotic plaques	Both	α-smooth muscle actin	Palmitate and atherosclerosis	p16, DNA damage	IL-1α, IL-1β, IL-6, MCP-1	Vascular senescence and atherosclerosis	Grootaert et al. (2021)
Vascular smooth muscle cells	Human atherosclerotic plaques	*In vitro*	α-smooth muscle actin	Atherosclerosis	SA-β-Gal, p16, p21, DNA damage	IL-1α, IL-6, MCP-1	Vascular senescence and atherosclerosis	Wang et al. (2015a)
Vascular smooth muscle cells	Human atherosclerotic plaques	*In vitro*	α-smooth muscle actin	Atherosclerosis	SA-β-Gal, p16, p21, telomere shortening, DNA damage		Vascular senescence and atherosclerosis	Matthews et al. (2006)
Vascular smooth muscle cells	Mouse aorta	*In vitro*	Isolation of VSMCs	Autophagy defection	P53, p21	TGF-β, MMP9, CXCL12	Vascular senescence and atherosclerosis	Grootaert et al. (2015)
Vascular smooth muscle cells	Mouse aorta	Both	Isolation of VSMCs, SM22α	Aging, Angiotensin II	SA-β-Gal p16, p21, p53		Vascular senescence and atherosclerosis	Miao et al. (2017)
Cardiac stem cells	Human stem cells	*In vitro*		Age differences	p16, SA-β-Gal	IL-6, IL-1β	Cardiac stem cell aging	Nakamura et al. (2017)
Cardiac stem cells	High glucose exposure	*In vitro*	c-kit positive cells	High glucose	p53, p16, SA-β-Gal		Stem cell dysfunction	Fu et al. (2019)
Cardiac stem cells	Mouse hearts	Both	c-kit positive cells	MI	p53, SA-β-Gal		Cardiac dysfunction	Toko et al. (2014)
Mesenchymal stem cells	Human stem cells	*In vitro*		Aging	ISA-β-Gal, p53, p21		Stem cell senescence	Hong et al. (2020)

Several studies correlate elevated sEH levels observed in aging with age-associated diseases and disorders. For example, age-related increased sEH protein expression and activity has been found in the brain (Nelson et al., 2014), heart (Jamieson et al., 2020; Yousef et al., 2024), intestines (Wang et al., 2023), liver (Wu et al., 2023), and kidney (Jamieson et al., 2020) in both human and murine models. In the senescence-accelerated mouse (SAMP8) model, increased levels of the oxylipin 9,10-DiHOME, a sEH-derived pro-inflammatory metabolite of linoleic acid, was associated with aging (Currais et al., 2015). Elevated sEH activity in the progression of biological aging is linked to the rapid hydrolysis of epoxylipids and accumulation of less potent or even pro-inflammatory diols metabolites (Jamieson et al., 2017a; Edin and Zeldin, 2021). Recent evidence suggests both sEH deletion and supplementation of epoxylipids are effective in reducing several senescence signaling pathways as well inducing SASP in aged mice (Griñán-Ferré et al., 2020; Zhang et al., 2020; Zhang et al., 2022; Wang et al., 2023; Zhang et al., 2023; Yousef et al., 2024). Further evidence demonstrated pharmacological inhibition of sEH prevented D-galactose-induced premature aging by decreasing senescent expression of p16, p21, and γH2AX (Zhang et al., 2023). *In vitro* experiments using a diseased lung model revealed that administration of PTUPB, a dual COX-2/sEH inhibitor, decreased the expression levels of p16(Ink4a) and p53-p21(Waf1/Cip1) (Zhang et al., 2020; Zhang et al., 2023). Moreover, 14,15-EET treatment alleviated endoplasmic reticulum stress and senescence in alveolar epithelial cells via antioxidant effects, suggesting that EETs serve as intrinsic molecules with potential anti-aging properties (Sun et al., 2016; Li et al., 2023). Additionally, sEH inhibition attenuated the expression of SASP-associated pro-inflammatory cytokines for example, the administration of sEH inhibitor drugs TPPU, AS-2586114, or UB-EV-52 to SAMP8 mice resulted in decreased Il-1β, CCL3, and TNF-α levels, as well as reduced oxidative stress markers in senescent mice (Griñán-Ferré et al., 2020; Yang et al., 2020; Jarne-Ferrer et al., 2022; Zhang et al., 2022).

While a connection between tissue aging and sEH activity has been documented, the precise mechanism by which sEH attenuates senescence remains unclear. Evidence indicates inhibiting sEH or increasing epoxylipid levels might improve the removal of senescent cells and subsequently slow down the aging process, acting as a senomorphic agents across the cellular environment. The mechanism(s) likely involves protecting mitochondrial function, thereby reducing cellular senescence.

## 4 The multifaceted role of mitochondria

**FIGURE 4:**
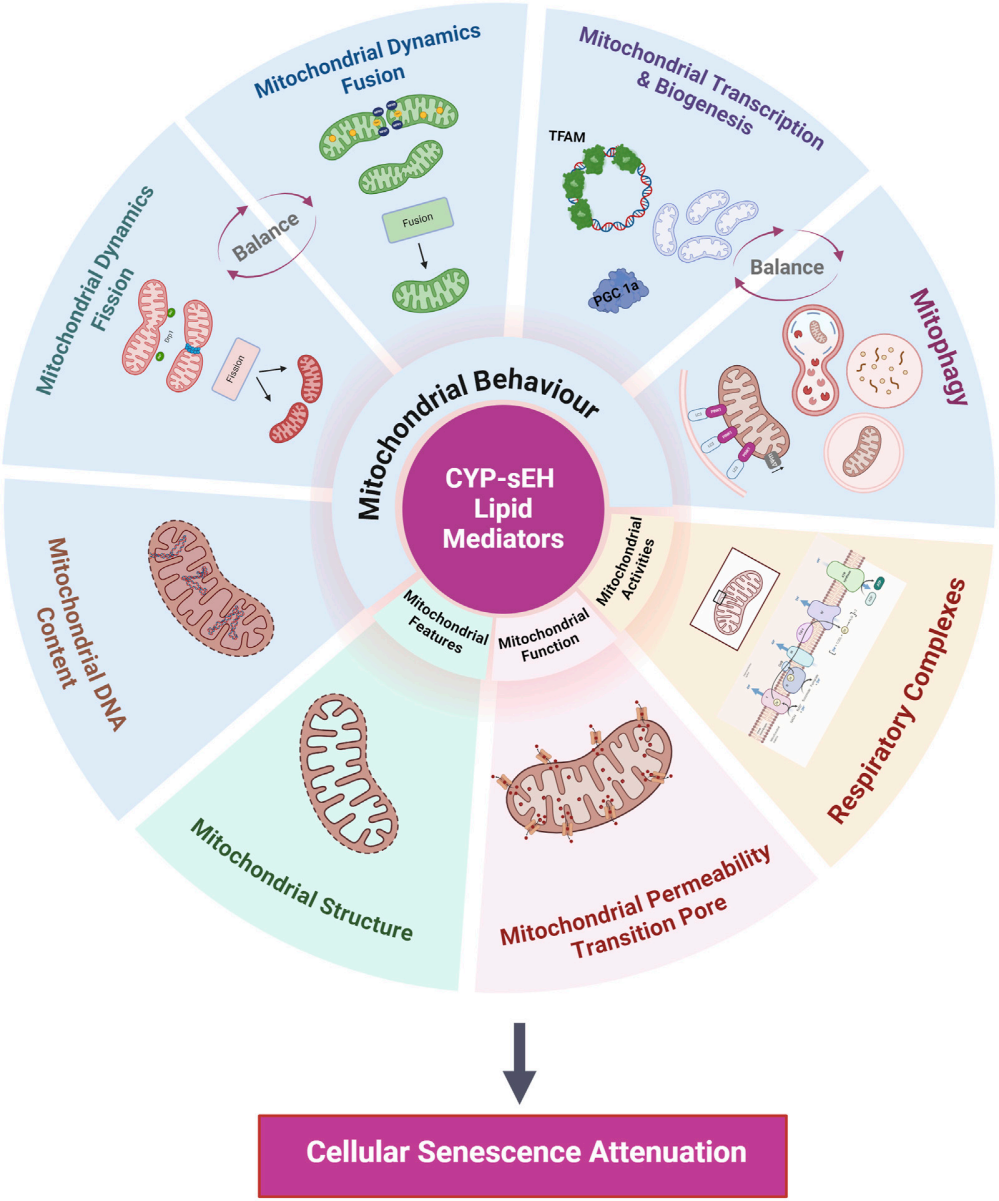
Summary of mitochondrial processes impacted by CYP-sEH metabolites. Proposed mitochondrial processes effected by CYP-sEH metabolites that potentially regulate cellular senescence include mitochondrial behaviours, features, function and activities. Created with BioRender.com.

Mitochondrial are multifunctional organelles that dynamically recalibrate their features, activities, functions, and behaviors based on endogenous and exogenous factors (Monzel et al., 2023). Mitochondrial impairments and adaptive recalibrations are closely interconnected with cellular senescence often influencing each other in a feedback loop to act as key drivers of aging and age-related diseases (Chapman et al., 2019). In the current discussion, we refer to the multifaceted biology of mitochondria dysfunction as changes in functions and behaviours that arise from various factors, including impaired dynamics, dysregulated biogenesis and/or mitophagy, mtDNA signalling and expression, reduced respiratory capacity, and mitochondrial structural changes (Monzel et al., 2023) (Figure 4).

The mitochondrial respiratory chain is a major source of ROS production, as electron leakage forms superoxide anions upon reaction with molecular oxygen (Sawyer and Colucci, 2000). Impairment in mitochondrial functions often result in increased superoxide production, which can cause extensive damage to both nuclear and mitochondrial DNA inducing cellular senescence as well increasing the expression of SASP (Correia-Melo et al., 2016; Nelson et al., 2018). Naturally, with aging and senescence, the efficiency of metabolic processes diminish, leading to a redox imbalance between oxidant formation and detoxification (Pagan et al., 2022). This imbalance results in free radical-induced damage to macromolecules, contributing to the aging process and the emergence of age-related diseases, including CVD (Jomova et al., 2023). Concurrently, aging tissues and senescent cells experience a decline in mitochondrial function through reduced respiratory capacity (Miwa et al., 2022). Thus, resulting in increased production of superoxide anions, hydroxyl radicals, and hydrogen peroxide, correlating with changes in the mitochondrial membrane potential (Trifunovic and Larsson, 2008). For instance, in the basal mitochondrial respiration state, high membrane potential drives excessive superoxide production, further exacerbating mitochondrial dysfunction in aging cells (Korshunov et al., 1997). Furthermore, the significance of electron transport chain (ETC) complexes in the loss of respiratory capacity with aging is notable, for example, function of complex I and ROS production depend on the integrity of its assembly, which declines with age (Desler et al., 2012). Interestingly, knockdown of a single complex I assembly factor can cause cell senescence (Miwa et al., 2014). As well, increased mitochondrial hydrogen peroxide levels can increase SASP either by directly activating NF-kB signaling or indirectly by causing DNA damage and a DNA damage response (DDR) (Rodier et al., 2009). ROS-mediated DNA damage results in DNA strand breaks, or oxidative damage, with telomeres appearing to be particularly sensitive to oxidation due to their guanine-rich regions (Grollman and Moriya, 1993). The mitochondrial superoxide levels produced in senescent cells exert both autocrine and paracrine effects, demonstrating a role in sustaining cellular senescence through a positive loop that continually generates DNA damage (Passos et al., 2010). Conversely, senescent cells with depleted mitochondria lose their pro-inflammatory and pro-oxidant phenotype (Correia-Melo et al., 2016).

### 4.1 Mitochondrial behaviours - dynamics

Mitochondria are highly dynamic cellular organelles that undergo fusion and fission events crucial for maintaining function, quality control and cellular homeostasis. The balance between fission and fusion plays a role in preserving a healthy pool of mitochondria within the cell. Fusion will merge individual mitochondria into interconnected networks, which mixes contents such as mtDNA, proteins and lipids. Key mitochondrial fusion proteins include mitofusin 1 and 2 (MFN-1 and 2) that facilitate the fusion process of the outer mitochondrial membrane along with optic atrophy 1 (OPA1) that have a role in cristae structure and regulate the fusion of the inner mitochondrial membrane (Youle and van der Bliek, 2012). Whereas mitochondrial fission segregates mitochondria into smaller units containing damaged proteins, destabilized membranes, and damaged mtDNA in preparation for removal via mitophagy as well distribute mitochondria during cell division (Pagliuso et al., 2018). Key proteins involved in the regulation of fission include dynamin-related protein 1 (DRP-1) and fission protein 1 (FIS1), which are recruited at specific sites on the mitochondrial membrane. Two distinct types of fission, mediated by DRP1, were identified: peripheral division, enabling damaged or misfolded proteins in mitochondria to be encapsulated into smaller mitochondria for mitophagy, and middle division, considered a way to increase mitochondrial mass (Kleele et al., 2021).

Simultaneous disruption of mitochondrial behaviors through fission and fusion processes can accelerate the accumulation of impaired mitochondria, which contributes to the progression of senescence within the aging heart (Dai and Rabinovitch, 2009). For instance, in senescent Hela cells, elongated and hyperfused mitochondrial networks have been associated with a reduced expression of the FIS1 protein (Lee et al., 2007). Reducing FIS1 expression has been demonstrated to increase ROS, in particular superoxide, nitric oxide and hypochlorous acid, production and trigger cellular senescence. Conversely, the overexpression of FIS1 has been shown to counteract mitochondrial elongation and reverse the senescent phenotype (Judge and Leeuwenburgh, 2007). Moreover, simultaneous downregulation of DRP1 and FIS1 proteins has been observed in aged mammals (Yoon et al., 2006; Lee et al., 2007; Mai et al., 2010). Inducing DRP1-mediated fission during mid-life in *Drosophila* has been shown to enhance mitochondrial respiratory function and structure, extend lifespan, and delay age-related pathologies (Rana et al., 2017). Conversely, studies indicate that mitochondrial fusion becomes compromised during aging, where a progressive reduction in MFN2 was associated with aging in skeletal muscles (Sebastián et al., 2016; Zeng et al., 2020; Kleele et al., 2021; Liu et al., 2021). Impaired mitochondrial fusion during aging, leads to decreased ATP production and accumulation of mutated mtDNA, which have been associated with an induction of senescence-associated pathways (Yoon et al., 2006; Lee et al., 2012).

### 4.2 Mitochondrial behaviours - mitophagy

Mitophagy is the specific autophagic breakdown of damaged mitochondria and is crucial for maintaining mitochondrial and cellular homeostasis (Chen et al., 2020). Following recruitment through various ubiquitination pathways, mitochondria are enveloped by autophagosomes and merged with lysosomes for subsequent degradation. Impaired mitophagy, results in the accumulation of damaged mitochondria and accelerated cellular senescence, as evident in aged rat and human studies (O’Leary et al., 2013; Dalle Pezze et al., 2014; Garcia-Prat et al., 2016; Chen et al., 2020). Mitophagy often declines with age because of several factors, such as lysosomal malfunction or lysosomal overload which prevents effective targeting of the autophagosomes. This is in turn results in the inadequate removal of damaged mitochondria (Kissova et al., 2004; D’Amico et al., 2019). Evidence suggests that in aged and senescent cells, lysosomes demonstrate reduced activity which results in accumulation of undegraded organelles (Sitte et al., 2001). When mitochondria are damaged, the PTEN-induced kinase 1 (PINK1) protein accumulates on the outer membrane leading to recruitment of the E3 ubiquitin ligase Parkin, which ubiquitinates various proteins on the outer mitochondrial membrane. This ubiquitination process facilitates autophagosome formation and the subsequent lysosomal removal of damaged mitochondria (Zimmermann et al., 2021). Comprised mitophagy can arise from defects in mitochondrial dynamics, the effectiveness of this process hinges on the organelles capacity to undergo fission and be eliminated (Twig and Shirihai, 2011). The efficiency of both fission and mitophagy can deteriorate with aging due to diminished expression of PINK1 (Bueno et al., 2015). In terms of senescence, it has been proposed the accumulation of the protein p53 in the cytosol leads to the sequestration of Parkin, hindering its translocation to the mitochondria thus serving to reinforce the senescence phenotype (Hoshino et al., 2013). A previous study indicates that the diminished translocation of Parkin to mitochondria leads to the buildup of damaged mitochondria and is linked to the initiation of senescence (Ahmad et al., 2015). In a similar model of senescence, Araya et al. (2019) found that knocking down Parkin resulted in impaired mitophagy and the induction of senescence, while its overexpression was adequate to trigger mitophagy and attenuate cellular senescence (Araya et al., 2019).

### 4.3 Mitochondrial DNA - expression and signalling

Mitochondria contain circular double stranded DNA limited to 16,569 base pairs organized in nucleoid-like structures within the mitochondrial matrix (Chinnery and Hudson, 2013). The mitochondrial genome exists as a multi-copy structure, with each cell containing hundreds to thousands of mtDNA copies that are subject to variations based on cell metabolism and exposure to stressors (Bonawitz et al., 2006). The regulation of mtDNA replication and transcription falls under the control of the peroxisome proliferator-activated receptor gamma coactivator 1-alpha (PGC-1α), a regulator of mitochondrial biogenesis (Scarpulla, 2011). Activated by phosphorylation or deacetylation, PGC-1α triggers the expression of transcription factors such as the nuclear respiratory factor (NRF) 1 and 2, and estrogen-related receptor-α, promoting the expression of mitochondrial transcription factors A, B1, and B2 (TFAM, TFB1M, and TFB2M) (Handschin and Spiegelman, 2006; Rebelo et al., 2011). TFAM plays a pivotal role in the regulation of mitochondrial biogenesis, binding to mtDNA and initiate mitochondrial transcription and replication (Picca and Lezza, 2015).

In response to stress, cells can release endogenous components into the extracellular space to initiate immune responses, collectively termed damage-associated molecular patterns (DAMPs). These DAMPs are recognized by pattern-recognition receptors, strongly expressed in immune cells but also present throughout the body, and they stimulate inflammatory responses (Dela Cruz and Kang, 2018; Grazioli and Pugin, 2018). The inflammatory responses during aging and age-related diseases may results from the innate immune response identifying damaged mitochondrial components. Notably, a subclass of DAMPs commonly referred to as mitochondrial DAMPs including cardiolipin, TFAM, ATP, succinate, cytochromeC, and mtDNA (Nakahira et al., 2015; Grazioli and Pugin, 2018). When in the cytosol, mtDNA serves as a DAMP, triggering inflammation, and tissue damage (Riley and Tait, 2020). The presence of circulating mtDNA gradually rises with advanced aging and has a closely linked relationship to inflammatory conditions (Pinti et al., 2014). The levels of circulating mtDNA to the cytosol or extracellular space align with the increased serum inflammatory markers and senescent cells (Pinti et al., 2014; Victorelli et al., 2023). Furthermore, circulating mtDNA has been detected in extracellular fluid following cell injuries such as acute MI, and sepsis (Nie et al., 2020).

Studies report a decrease in mtDNA content with age, with older individuals experiencing more significant decline (Mengel-From et al., 2014; Knez et al., 2016). Lower mtDNA content in older individuals is associated with mortality, cognitive decline, and reduced physical performance (Mengel-From et al., 2014). An early hypothesis suggested that the accumulation of mutated mtDNA with age might directly contribute to the decline in mitochondrial function (Kujoth et al., 2005; Shimada et al., 2012). The mitochondrial theory of aging suggests that mtDNA mutations accumulate over time due to less active repair mechanisms when compared to nuclear DNA (nDNA) and proximity to ETC, which is a major source of ROS (La Morgia et al., 2020; Wolf, 2021). Subsequently, mtDNA has a relatively high mutation rate resulting in extensive polymorphism which can disrupt ETC assembly, transmembrane potential dispersion, and increase ROS production (Judge and Leeuwenburgh, 2007). Studies indicate mtDNA mutations increase with age and can be observed in various tissues and organs (Srivastava, 2017). Other studies report that despite the accumulation of mtDNA mutations leading to premature aging phenotypes, there was no corresponding increase in ROS levels or oxidative stress. This suggests that respiratory chain dysfunction, rather than increased oxidative stress, was a key driver of aging (Trifunovic et al., 2005).

### 4.4 Mitochondrial activities - NAD+/NADH ratio and sirtuins

The silent mating-type information regulation 2 homologs or sirtuins (SIRTs) are a group of nicotinamide adenine dinucleotide (NAD+)-dependent deacylases that include seven mammalian family members identified as SIRT1 to SIRT7. SIRT1, -6, and -7 are mainly localized in the nucleus, while SIRT1 often localizes to the cytoplasm. SIRT2 is mainly found in the cytoplasm, with specific splice isoforms found in the nucleus under certain conditions. SIRT3-5 primarily reside within mitochondria (Dang, 2014). The SIRT family is heavily involved in intracellular signalling and plays various roles in maintaining cardiac homeostasis, metabolism and aging (Wu et al., 2022). As the aging process progresses, studies have found a decrease in both SIRT1 and NAD+ levels in the liver and skeletal muscle tissues in aged mice (Yoshino et al., 2011; Mouchiroud et al., 2013; Imai and Guarente, 2014). Moreover, declines in NAD+ levels are closely linked to onset of aging related conditions such as obesity, muscle loss and diabetes (Yoshino et al., 2011; Canto et al., 2012; Gomes et al., 2013; Zhang et al., 2016). In contrast, elevated expression of SIRT1 has been shown to suppress the transcription of SASP markers in cardiomyocytes and endothelial cells (Zheng et al., 2012; Vassallo et al., 2014; Man et al., 2019). Mitochondrial SIRT3 plays a crucial role in eliminating intracellular ROS and maintaining oxidative metabolism through MnSOD deacetylation (Yu et al., 2012; Parodi-Rullán et al., 2018; Sun et al., 2018). Depletion of SIRT3 is marked by heightened oxidative stress and senescence markers. Recent findings indicate that stressed or damaged cells can restore NAD+ levels through a cytosolic complex of enzymes that transfers electrons from NADH to NADP+. This reaction is potentially important to preventing cellular senescence (Igelmann et al., 2021). Crucially, decreased NAD+ levels are linked to dysregulated mitochondrial activities during aging. Moreover, a lowered NAD+/NADH ratio has been implicated in the regulation of the SASP (Velarde et al., 2012; Nacarelli et al., 2016). Findings from human fibroblasts showed that SIRT3 overexpression antagonized premature senescence and reduced β-gal and p16 expression (Zhang et al., 2013). SIRT3 is involved in antagonizing cellular senescence was linked to improving mitochondria homeostasis and mitophagy (Watroba and Szukiewicz, 2016; Guo et al., 2021). For these reasons, the modulation of SIRT activity and NAD+ levels and their associated molecular pathways have been investigated as potential targets for anti-aging therapies (Grabowska et al., 2017).

### 4.5 Mitochondrial functions - mitochondrial permeability transition pore

The mitochondrial permeability transition pore (mPTP) is a transmembrane protein that is responsible for mitochondrial permeability which enables the free entry of molecules with a molecular weight of up to 1.5 kDa (Halestrap and Richardson, 2015). Opening of the pore which results in the internal accumulation of molecules, coupled with oxidative stress, eventually leads to mitochondrial swelling, dysfunction, irreversible ATP loss, and a sustained loss of mitochondrial membrane potential (Δψm) (Halestrap, 2010). Maintaining an electrochemical gradient is crucial for ATP production, and the collapse of the mitochondrial membrane potential (Δψm) is associated with mPTP opening (Levraut et al., 2003). Shared characteristics of aged tissues and senescent cells include the elevated concentration of mitochondrial calcium and ROS, molecules which stimulate the opening of the mPTP (Ziegler et al., 2015). Increased mPTP opening is marked by the reduction of mitochondrial membrane potential and release of mitochondrial Calcium and cytochrome C, thus increased ROS production (Baines et al., 2005). Evidence suggests that the mPTP can open in two distinct pathways, permanently or transiently. Persistent opening leads to cell death, whereas temporary activation can have beneficial effects (Pastorino et al., 1999). Transient activation permits the release or exchange of calcium, ROS or other molecules between the mitochondrial matrix and cytosol. The combination of high Calcium, high ROS, and low NAD+ in aged and senescent cells increases the likelihood of mPTP opening (Pastorino et al., 1999). In most diseases associated with cellular senescence, an increase in mPTP activation results in extensive toxicity and cell death (Mather and Rottenberg, 2000). In aged mice, an increased susceptibility to mPTP opening has been observed in the brain and liver (Goodell and Cortopassi, 1998; Mather and Rottenberg, 2000). Similarly, research on muscles from aged humans and rats has indicated reduced mitochondrial calcium retention capacity and increased sensitization of mPTP opening, leading to apoptosis (Gouspillou et al., 2014). Dysregulation of mitochondrial features and functions in aging tissues and senescent fibroblasts is characterized by a decrease in respiratory capacity and a decrease in the mitochondrial membrane potential (Δψm) (Passos et al., 2007; Correia-Melo et al., 2016; Rizza et al., 2018).

## 5 Protective role of epoxylipids

There is growing interest in therapeutically targeting mitochondria with the goal to stop, reverse, or slow down the pace of cellular deterioration and damage accumulation with age. Two present categories of senotherapies include the “senolytic” therapies, which selectively induce the death of senescent cells, and “senomorphic” therapies, which dampen the SASP components without impacting cell viability. Treatments that indirectly enhance the features, activities, functions, and behaviors of mitochondria can successfully reduce cellular senescence and increase organismal health. However, finding interventions to improve mitochondria in senescent cells is challenging due to the complexity and variability of mitochondrial biological processes. Emerging evidence suggests epoxylipids interact with mitochondria to improve age-related effects across different tissues and species. Numerous *in vivo* and *ex vivo* studies demonstrate epoxylipids enhance cardiac functional recovery following injury by protecting mitochondria (Katragadda et al., 2009; Batchu et al., 2012; Akhnokh et al., 2016; El-Sikhry et al., 2016; Jamieson et al., 2017a; Samokhvalov et al., 2018; Darwesh et al., 2019b; Keshavarz-Bahaghighat et al., 2020).

Mitochondrial-mediated events in aged hearts have been associated with the decline in overall cardiac function. Research from numerous studies indicate either inhibiting sEH to increase endogenous epoxylipid levels or direct administration of epoxylipids can positively modulate mitochondrial dynamics and protect against cellular damage (Scarpulla, 2011; Waldman et al., 2016; Wiley et al., 2016; Cao et al., 2017; Liu et al., 2018; Darwesh et al., 2019a). Inhibiting sEH has been observed to regulate the mitochondrial fusion-to-fission ratio by increasing Mfn-1 expression, which correlated with elevated ATP production and reduced oxidative stress (Liu et al., 2018; Darwesh et al., 2019a). Further, studies have shown that epoxylipids exert cytoprotective properties by increasing the expression of PGC-1α, a regulator of mitochondrial biogenesis (Scarpulla, 2011; Waldman et al., 2016; Wiley et al., 2016; Cao et al., 2017). In obesity-induced cardiomyopathy, EETs increased expression of PGC-1, Mfn2 and MnSOD, shedding additional light on their role in maintaining mitochondrial biogenesis (Cao et al., 2017). Activation of mitophagy removes damaged mitochondria and reduces production of ROS such as superoxide anion, hydrogen peroxide, and hydroxyl radical, to promote cell survival (De Gaetano et al., 2021). Jiang et al., found that inhibition of sEH with *t-*AUCB enhanced PINK1/Parkin-mediated mitophagy in the kidneys by increased formation of autophagosomes and fusion of autophagosome-lysosomes thus preserving kidney damage (Jiang et al., 2020). Furthermore, administration of 14,15-EET enhanced LC3-II expression and autophagosome generation in cardiac cells via AMPK activation (Samokhvalov et al., 2013), where AMPK leads to activation of mitophagy (Seabright et al., 2020). Moreover, in human cerebral microvascular endothelial cells, 14,15-EET regulated mitophagy and protected neuronal function against reperfusion induced injury (Qu et al., 2022). In contrast, recent study showed that 12,13-DiHOME, a diol metabolite, contributes to the pathophysiological immune response by altering mitophagy process in macrophage-like cell line (Valencia et al., 2024). Thus, the beneficial effects of sEH inhibition might be driven not only by increased epoxylipids, but also the reduction of diol metabolites. However, further exploration into the mechanisms underlying epoxylipid bioactivity and mitochondria in age-related disease pathogenesis are needed.

sEH genetic deletion has been shown to maintain mitochondrial electron transport complex activity and ATP generation in isolated murine cardiac fibers subjected to LAD in both young and aged mice (Jamieson et al., 2017b; Jamieson et al., 2021). In addition, sEH deletion preserved cardiac mitochondrial features by maintaining mtDNA mass content in aged female mice (Yousef et al., 2024) and maintained mitochondrial activities such as preserving electron chain enzymatic activity in LPS challenged mice (Samokhvalov et al., 2018). Furthermore, treatment of HL-1 cells or neonatal cardiomyocytes with UA-8, an EET mimetic with sEH inhibitory properties, enhanced the enzymatic activities of key mitochondrial respiratory chain proteins, including citrate synthase, succinate dehydrogenase, and cytochrome C oxidase (Samokhvalov et al., 2013). Additionally, UA-8 preserved mitochondrial respiratory control ratio and prevented the increase in the ADP/ATP ratio caused by starvation induced cell death, highlighting the role of epoxylipids in maintaining mitochondrial activities and functions like oxidative phosphorylation and ATP synthesis (El-Sikhry et al., 2016). Both endogenous 19,20-EDP, a CYP-derived N-3 PUFA metabolite, and a synthetic structural analog SA-22 exhibited cardioprotective benefit against hypoxia-reoxygenation injury in several *in vitro* models. Importantly, the salutary effects of these compounds were attributed to the preservation of mitochondrial activities and respiratory function, dependent upon sirtuin activity (Akhnokh et al., 2016; Jamieson et al., 2020; Kranrod et al., 2024). Sirtuin 3 (SIRT3), a NAD-dependent deacetylase primarily located in mitochondria, has emerged as a crucial mediator in age-related cardiovascular physiology by modulating mitochondrial oxidative stress through MnSOD deacetylation (Parodi-Rullán et al., 2018; Sun et al., 2018). Cardiomyocytes lacking SIRT3 exhibit age-dependent mitochondrial swelling and accelerated signs of cardiac aging, including myocardial hypertrophy and accumulated fibrotic tissue (Hafner et al., 2010). Intriguingly, while the cardiac expression of sEH increases significantly during aging, the genetic deletion of sEH mitigates the age-related decline in SIRT3 activity in female mice (Jamieson et al., 2020). This effect correlates with elevated levels of active mitochondrial MnSOD, promoting improved overall cardiac function and suggesting the preservation of mitochondria in aged mice (Jamieson et al., 2020).

Many studies have observed the preservation of mitochondrial membrane potential with sEH inhibition and/or EET administration. Experiments have demonstrated that exogenous EETs can delay the dissipation of Δψm and the opening of the mPTP in rat cardiomyocytes and H9c2 cells. Furthermore, this effect was nullified with co-treatment using the EET antagonist 14,15-epoxyeicosa-5(Z)-enoic acid (14,15-EEZE) (Katragadda et al., 2009; Batchu et al., 2012). Supportive data from non-cardiac cells show that EETs exert mitoprotective effects, such as in rat hippocampal astrocytes, where 11,12- and 14,15-EET attenuated mitochondrial fragmentation, preserved Δψm, and improved respiration after treatment with amyloid-β protein (Sarkar et al., 2014). Additionally, inhibition of endogenous EET production using the selective epoxygenase inhibitor MS-PPOH disrupted mitochondrial ATP generation, increased hydrogen peroxide production, and induced mitochondrial depolarization and fragmentation in cultured hippocampal astrocytes (Sarkar et al., 2014). However, it remains unclear how EET-mediated events preserve Δψm and whether this preservation is achieved through a direct or indirect effect on the mitochondria. These findings suggest a role for EETs in minimizing the loss of Δψm and limiting mPTP opening, thereby contributing to overall cardiac protection under stress. Additionally, young and aged sEH null mice exhibited preserved mitochondrial ultrastructure following MI, characterized by improved cristae density and organization (Akhnokh et al., 2016; Jamieson et al., 2017b). In a study, it was found that 14,15-EET enhanced the expression of nuclear gene-encoded mitochondrial proteins, such as PGC-1α, NRF-1, and TFAM, thereby contributing to the preservation of mitochondrial DNA (mtDNA) (Wang et al., 2014). Genetic deletion and pharmacological inhibition of sEH resulted in increased mtDNA and normalized TFAM expression in aged female hearts subjected to LPS endotoxin challenge (Yousef et al., 2024).

## 6 Conclusion

CYP-derived epoxylipids are an emerging class of protective lipid mediators that play a critical role in aging and CVD; however, their exact mechanism of action remains unknown. Interestingly, evidence indicates increased expression of sEH correlates with decreased epoxylipid levels in some aged tissues. In an aging heart, changing mitochondrial biology can result in a senescent phenotype that contributes to a decline in cardiac function and increase susceptibility to CVD. We propose that CYP-derived epoxylipids mediate effects in mitochondria that can regulate cellular senescence and suppress the pro-inflammatory SASP response. Our current understanding suggests they effect mitochondrial biology through several proposed molecular mechanisms (Monzel et al., 2023). These involve maintaining mitochondrial morphology and ultrastructure (mitochondrial features), limiting oxidative stress and enhancing respiration by maintaining electron transport chain enzyme activities (mitochondrial activities), preserving mitochondrial membrane potential and delaying the opening of the mPTP (mitochondrial function), improving mitochondrial fission and fusion dynamics and maintaining mitochondrial DNA content (mitochondrial behaviour). However, it is important to consider sEH-derived metabolites generated within a cell can potentially have adverse effects as well. Thus, highlighting our limited understanding of the role the metabolites generated by CYP-sEH metabolism have within cells. Furthermore, research addressing knowledge gaps in our understanding of the pathophysiology of the aging heart and the complexity of mitochondrial biology will be critical, notably in relation to senescence. In conclusion, emerging evidence suggests a role for CYP-sEH derived metabolites of N-3 and N-6 PUFA in regulating mitochondria and cellular senescence, which can impact the aging heart.

## Glossary

CVD: Cardiovascular Diseases
CYP: Cytochrome P450
PUFAs: Polyunsaturated Fatty Acids
RCR: Respiratory Control Ratio
ADP: Adenosine Diphosphate
ATP: Adenosine Triphosphate
SIPS: Stress-Induced Premature Senescence
ALA: Alpha Linolenic Acid
EPA: Eicosapentaenoic Acid
DHA: Docosahexaenoic Acid
LA: Linoleic Acid
AA: Arachidonic Acid
COX: Cyclooxygenases
LOX: Lipoxygenases
EEQ: Epoxyeicosatetraenoic Acid
EDP: Epoxydocosapentaenoic Acid
EpOME: Epoxyoctadecenoic Acid
EET: Epoxyeicosatrienoic Acid
sEH: Soluble Epoxide Hydrolase
mEH: Microsomal Epoxide Hydrolase
DHEQ: Dihydroxyeicosatetraenoic Acid
DHDP: Dihydroxydocosapentaenoic Acid
DiHOME: Dihydroxyoctadecenoic Acid
DHET: Dihydroxyeicosatrienoic Acid
CYP2J2: Cytochrome P450 2J2
c-AUCB: cis-4-[4-(3-Adamantan-1-yl-ureido) cyclohexyloxy] benzoic Acid
sEHi: Soluble Epoxide Hydrolase Inhibitor
LPS: Lipopolysaccharide
SASP: Senescence-Associated Secretory Phenotype
β-gal: Beta-Galactosidase
p53: Tumor Suppressor Protein p53
ATM: Ataxia Telangiectasia Mutated
ATR: Ataxia Telangiectasia and Rad3 Related
CHK1: Checkpoint Kinase 1
CHK2: Checkpoint Kinase 2
p21/CDKN1A: Cyclin-Dependent Kinase Inhibitor 1A
p16INK4a: Cyclin-Dependent Kinase Inhibitor 2A
CDK4: Cyclin-Dependent Kinase 4
GDF15: Growth Differentiation Factor 15
IL-6: Interleukin 6
IL-8: Interleukin 8
IL-11: Interleukin 11
IL-1α: Interleukin 1 Alpha
IL-1β: Interleukin 1 Beta
MCP-1: Monocyte Chemoattractant Protein 1
TNF-α: Tumor Necrosis Factor Alpha
TGFβ: Transforming Growth Factor Beta
NF-kB: Nuclear Factor kappa B
9,10-DiHOME: 9,10-Dihydroxyoctadecenoic Acid
D-gal: D-galactose
γH2AX: Gamma-H2A Histone Family Member X
DDR: DNA Damage Response
ETC: Electron Transport Chain
mtROS: Mitochondrial Reactive Oxygen Species
MFN-1: Mitofusin 1
MFN-2: Mitofusin 2
OPA1: Optic Atrophy 1
DRP-1: Dynamin-Related Protein 1
FIS1: Fission Protein 1
PINK1: PTEN-Induced Kinase 1
TFAM: Mitochondrial Transcription Factor A
NRF: Nuclear Respiratory Factor
DAMPs: Damage-Associated Molecular Patterns
NAD+: Nicotinamide Adenine Dinucleotide
SIRTs: Sirtuins
MnSOD: Manganese Superoxide Dismutase
mPTP: Mitochondrial Permeability Transition Pore
Δψm: Mitochondrial Membrane Potential
SIRT3: Sirtuin 3

## References

[B1] Ahmad T. Sundar I. K. Lerner C. A. Gerloff J. Tormos A. M. Yao H. (2015). Impaired mitophagy leads to cigarette smoke stress-induced cellular senescence: implications for chronic obstructive pulmonary disease. FASEB J. 29, 2912–2929. 10.1096/fj.14-268276 25792665 PMC4478793

[B2] Akhnokh M. K. Yang F. H. Samokhvalov V. Jamieson K. L. Cho W. J. Wagg C. (2016). Inhibition of soluble epoxide hydrolase limits mitochondrial damage and preserves function following ischemic injury. Front. Pharmacol. 7, 133. 10.3389/fphar.2016.00133 27375480 PMC4896112

[B3] Aliwarga T. Evangelista E. A. Sotoodehnia N. Lemaitre R. N. Totah R. A. (2018). Regulation of CYP2J2 and EET levels in cardiac disease and diabetes. Int. J. Mol. Sci. 19, 1916. 10.3390/ijms19071916 29966295 PMC6073148

[B4] Amaya-Montoya M. Pérez-Londoño A. Guatibonza-García V. Vargas-Villanueva A. Mendivil C. O. (2020). Cellular senescence as a therapeutic target for age-related diseases: a review. Adv. Ther. 37, 1407–1424. 10.1007/s12325-020-01287-0 32185730 PMC7140757

[B5] Ander B. P. Dupasquier C. M. Prociuk M. A. Pierce G. N. (2003). Polyunsaturated fatty acids and their effects on cardiovascular disease. Exp. Clin. Cardiol. 8, 164–172. 19649216 PMC2719153

[B6] Anderson R. Lagnado A. Maggiorani D. Walaszczyk A. Dookun E. Chapman J. (2019). Length-independent telomere damage drives post-mitotic cardiomyocyte senescence. EMBO J. 38, e100492. 10.15252/embj.2018100492 30737259 PMC6396144

[B7] Araya J. Tsubouchi K. Sato N. Ito S. Minagawa S. Hara H. (2019). PRKN-regulated mitophagy and cellular senescence during COPD pathogenesis. Autophagy 15, 510–526. 10.1080/15548627.2018.1532259 30290714 PMC6351145

[B8] Baines C. P. Kaiser R. A. Purcell N. H. Blair N. S. Osinska H. Hambleton M. A. (2005). Loss of cyclophilin D reveals a critical role for mitochondrial permeability transition in cell death. Nature 434, 658–662. 10.1038/nature03434 15800627

[B9] Baker D. J. Childs B. G. Durik M. Wijers M. E. Sieben C. J. Zhong J. (2016). Naturally occurring p16(Ink4a)-positive cells shorten healthy lifespan. Nature 530, 184–189. 10.1038/nature16932 26840489 PMC4845101

[B10] Balaban R. S. Nemoto S. Finkel T. (2005). Mitochondria, oxidants, and aging. Cell 120, 483–495. 10.1016/j.cell.2005.02.001 15734681

[B11] Ball A. J. Levine F. (2005). Telomere-independent cellular senescence in human fetal cardiomyocytes. Aging Cell 4, 21–30. 10.1111/j.1474-9728.2004.00137.x 15659210

[B12] Batchu S. N. Lee S. B. Qadhi R. S. Chaudhary K. R. El-Sikhry H. Kodela R. (2011). Cardioprotective effect of a dual acting epoxyeicosatrienoic acid analogue towards ischaemia reperfusion injury. Br. J. Pharmacol. 162, 897–907. 10.1111/j.1476-5381.2010.01093.x 21039415 PMC3042200

[B13] Batchu S. N. Lee S. B. Samokhvalov V. Chaudhary K. R. El-Sikhry H. Weldon S. M. (2012). Novel soluble epoxide hydrolase inhibitor protects mitochondrial function following stress. Can. J. Physiol. Pharmacol. 90, 811–823. 10.1139/y2012-082 22624559

[B14] Bellien J. Joannides R. (2013). Epoxyeicosatrienoic acid pathway in human health and diseases. J. Cardiovasc. Pharmacol. 61, 188–196. 10.1097/FJC.0b013e318273b007 23011468

[B15] Birks E. J. Latif N. Enesa K. Folkvang T. Luong L. A. Sarathchandra P. (2008). Elevated p53 expression is associated with dysregulation of the ubiquitin-proteasome system in dilated cardiomyopathy. Cardiovasc. Res. 79, 472–480. 10.1093/cvr/cvn083 18375498

[B16] Blazkova H. Krejcikova K. Moudry P. Frisan T. Hodny Z. Bartek J. (2010). Bacterial intoxication evokes cellular senescence with persistent DNA damage and cytokine signalling. J. Cell Mol. Med. 14, 357–367. 10.1111/j.1582-4934.2009.00862.x 19650831 PMC3837606

[B17] Bode A. M. Dong Z. (2004). Post-translational modification of p53 in tumorigenesis. Nat. Rev. Cancer 4, 793–805. 10.1038/nrc1455 15510160

[B18] Bonawitz N. D. Clayton D. A. Shadel G. S. (2006). Initiation and beyond: multiple functions of the human mitochondrial transcription machinery. Mol. Cell 24, 813–825. 10.1016/j.molcel.2006.11.024 17189185

[B19] Bueno M. Lai Y. C. Romero Y. Brands J. St Croix C. M. Kamga C. (2015). PINK1 deficiency impairs mitochondrial homeostasis and promotes lung fibrosis. J. Clin. Invest. 125, 521–538. 10.1172/JCI74942 25562319 PMC4319413

[B20] Cai Y. Liu H. Song E. Wang L. Xu J. He Y. (2021). Deficiency of telomere-associated repressor activator protein 1 precipitates cardiac aging in mice *via* p53/PPARα signaling. Theranostics 11, 4710–4727. 10.7150/thno.51739 33754023 PMC7978321

[B21] Caligiuri S. P. B. Aukema H. M. Ravandi A. Lavallee R. Guzman R. Pierce G. N. (2017a). Specific plasma oxylipins increase the odds of cardiovascular and cerebrovascular events in patients with peripheral artery disease. Can. J. Physiol. Pharmacol. 95, 961–968. 10.1139/cjpp-2016-0615 28714336

[B22] Caligiuri S. P. B. Parikh M. Stamenkovic A. Pierce G. N. Aukema H. M. (2017b). Dietary modulation of oxylipins in cardiovascular disease and aging. Am. J. Physiol. Heart Circ. Physiol. 313, H903–H918. 10.1152/ajpheart.00201.2017 28801523

[B23] Campbell W. B. Imig J. D. Schmitz J. M. Falck J. R. (2017). Orally active epoxyeicosatrienoic acid analogs. J. Cardiovasc. Pharmacol. 70, 211–224. 10.1097/FJC.0000000000000523 28937442 PMC5673125

[B24] Canto C. Houtkooper R. H. Pirinen E. Youn D. Y. Oosterveer M. H. Cen Y. (2012). The NAD(+) precursor nicotinamide riboside enhances oxidative metabolism and protects against high-fat diet-induced obesity. Cell Metab. 15, 838–847. 10.1016/j.cmet.2012.04.022 22682224 PMC3616313

[B25] Cao J. Singh S. P. McClung J. A. Joseph G. Vanella L. Barbagallo I. (2017). EET intervention on Wnt1, NOV, and HO-1 signaling prevents obesity-induced cardiomyopathy in obese mice. Am. J. Physiol. Heart Circ. Physiol. 313, H368–H380. 10.1152/ajpheart.00093.2017 28576832 PMC5582926

[B26] Cervenka L. Huskova Z. Kopkan L. Kikerlova S. Sedlakova L. Vanourkova Z. (2018). Two pharmacological epoxyeicosatrienoic acid-enhancing therapies are effectively antihypertensive and reduce the severity of ischemic arrhythmias in rats with angiotensin II-dependent hypertension. J. Hypertens. 36, 1326–1341. 10.1097/HJH.0000000000001708 29570510 PMC7375140

[B27] Chang E. Harley C. B. (1995). Telomere length and replicative aging in human vascular tissues. Proc. Natl. Acad. Sci. U. S. A. 92, 11190–11194. 10.1073/pnas.92.24.11190 7479963 PMC40597

[B28] Chapman J. Fielder E. Passos J. F. (2019). Mitochondrial dysfunction and cell senescence: deciphering a complex relationship. FEBS Lett. 593, 1566–1579. 10.1002/1873-3468.13498 31211858

[B29] Chaudhary K. R. Abukhashim M. Hwang S. H. Hammock B. D. Seubert J. M. (2010). Inhibition of soluble epoxide hydrolase by trans-4- [4-(3-adamantan-1-yl-ureido)-cyclohexyloxy]-benzoic acid is protective against ischemia–reperfusion injury. J. Cardiovasc. Pharmacol. 55, 67–73. 10.1097/FJC.0b013e3181c37d69 19834332 PMC2824072

[B30] Chen G. Kroemer G. Kepp O. (2020). Mitophagy: an emerging role in aging and age-associated diseases. Front. Cell Dev. Biol. 8, 200. 10.3389/fcell.2020.00200 32274386 PMC7113588

[B31] Chen T. Liang Q. Xu J. Zhang Y. Zhang Y. Mo L. (2021). MiR-665 regulates vascular smooth muscle cell senescence by interacting with LncRNA GAS5/SDC1. Front. Cell Dev. Biol. 9, 700006. 10.3389/fcell.2021.700006 34386495 PMC8353444

[B32] Cheng S. Fernandes V. R. Bluemke D. A. McClelland R. L. Kronmal R. A. Lima J. A. (2009). Age-related left ventricular remodeling and associated risk for cardiovascular outcomes: the Multi-Ethnic Study of Atherosclerosis. Circ. Cardiovasc. Imaging 2, 191–198. 10.1161/CIRCIMAGING.108.819938 19808592 PMC2744970

[B33] Childs B. G. Durik M. Baker D. J. van Deursen J. M. (2015). Cellular senescence in aging and age-related disease: from mechanisms to therapy. Nat. Med. 21, 1424–1435. 10.1038/nm.4000 26646499 PMC4748967

[B34] Chimenti C. Kajstura J. Torella D. Urbanek K. Heleniak H. Colussi C. (2003). Senescence and death of primitive cells and myocytes lead to premature cardiac aging and heart failure. Circ. Res. 93, 604–613. 10.1161/01.RES.0000093985.76901.AF 12958145

[B35] Chinnery P. F. Hudson G. (2013). Mitochondrial genetics. Br. Med. Bull. 106, 135–159. 10.1093/bmb/ldt017 23704099 PMC3675899

[B36] Coppe J. P. Patil C. K. Rodier F. Sun Y. Munoz D. P. Goldstein J. (2008). Senescence-associated secretory phenotypes reveal cell-nonautonomous functions of oncogenic RAS and the p53 tumor suppressor. PLoS Biol. 6, 2853–2868. 10.1371/journal.pbio.0060301 19053174 PMC2592359

[B37] Correia-Melo C. Marques F. D. Anderson R. Hewitt G. Hewitt R. Cole J. (2016). Mitochondria are required for pro-ageing features of the senescent phenotype. EMBO J. 35, 724–742. 10.15252/embj.201592862 26848154 PMC4818766

[B38] Cui S. Xue L. Yang F. Dai S. Han Z. Liu K. (2018). Postinfarction hearts are protected by premature senescent cardiomyocytes via GATA 4-dependent CCN 1 secretion. J. Am. Heart Assoc. 7, e009111. 10.1161/JAHA.118.009111 30371213 PMC6222958

[B39] Cunnane S. C. (2003). Problems with essential fatty acids: time for a new paradigm? Prog. Lipid Res. 42, 544–568. 10.1016/s0163-7827(03)00038-9 14559071

[B40] Currais A. Goldberg J. Farrokhi C. Chang M. Prior M. Dargusch R. (2015). A comprehensive multiomics approach toward understanding the relationship between aging and dementia. Aging (Albany NY) 7, 937–955. 10.18632/aging.100838 26564964 PMC4694064

[B41] Dai D. F. Rabinovitch P. S. (2009). Cardiac aging in mice and humans: the role of mitochondrial oxidative stress. Trends Cardiovasc Med. 19, 213–220. 10.1016/j.tcm.2009.12.004 20382344 PMC2858758

[B42] Dalle Pezze P. Nelson G. Otten E. G. Korolchuk V. I. Kirkwood T. B. von Zglinicki T. (2014). Dynamic modelling of pathways to cellular senescence reveals strategies for targeted interventions. PLoS Comput. Biol. 10, e1003728. 10.1371/journal.pcbi.1003728 25166345 PMC4159174

[B43] D’Amico D. Mottis A. Potenza F. Sorrentino V. Li H. Romani M. (2019). The RNA-binding protein PUM2 impairs mitochondrial dynamics and mitophagy during aging. Mol. Cell 73, 775–787. 10.1016/j.molcel.2018.11.034 30642763 PMC6396316

[B44] Dang W. (2014). The controversial world of sirtuins. Drug Discov. Today Technol. 12, e9–e17. 10.1016/j.ddtec.2012.08.003 25027380 PMC4101544

[B45] Darwesh A. M. Jamieson K. L. Wang C. Samokhvalov V. Seubert J. M. (2019a). Cardioprotective effects of CYP-derived epoxy metabolites of docosahexaenoic acid involve limiting NLRP3 inflammasome activation (1). Can. J. Physiol. Pharmacol. 97, 544–556. 10.1139/cjpp-2018-0480 30326194

[B46] Darwesh A. M. Keshavarz-Bahaghighat H. Jamieson K. L. Seubert J. M. (2019b). Genetic deletion or pharmacological inhibition of soluble epoxide hydrolase ameliorates cardiac ischemia/reperfusion injury by attenuating NLRP3 inflammasome activation. Int. J. Mol. Sci. 20, 3502. 10.3390/ijms20143502 31319469 PMC6678157

[B47] De Gaetano A. Gibellini L. Zanini G. Nasi M. Cossarizza A. Pinti M. (2021). Mitophagy and oxidative stress: the role of aging. Antioxidants (Basel) 10, 794. 10.3390/antiox10050794 34067882 PMC8156559

[B48] Dela Cruz C. S. Kang M. J. (2018). Mitochondrial dysfunction and damage associated molecular patterns (DAMPs) in chronic inflammatory diseases. Mitochondrion 41, 37–44. 10.1016/j.mito.2017.12.001 29221810 PMC5988941

[B49] Desler C. Hansen T. L. Frederiksen J. B. Marcker M. L. Singh K. K. Juel Rasmussen L. (2012). Is there a link between mitochondrial reserve respiratory capacity and aging? J. aging Res. 2012, 192503. 10.1155/2012/192503 22720157 PMC3375017

[B50] Di Micco R. Krizhanovsky V. Baker D. d'Adda di Fagagna F. (2021). Cellular senescence in ageing: from mechanisms to therapeutic opportunities. Nat. Rev. Mol. Cell Biol. 22, 75–95. 10.1038/s41580-020-00314-w 33328614 PMC8344376

[B51] Dimri G. P. Lee X. Basile G. Acosta M. Scott G. Roskelley C. (1995). A biomarker that identifies senescent human cells in culture and in aging skin *in vivo* . Proc. Natl. Acad. Sci. 92, 9363–9367. 10.1073/pnas.92.20.9363 7568133 PMC40985

[B52] Doleželová Š. Jíchová Š. Husková Z. Vojtíšková A. Kujal P. Hošková L. (2016). Progression of hypertension and kidney disease in aging fawn-hooded rats is mediated by enhanced influence of renin–angiotensin system and suppression of nitric oxide system and epoxyeicosanoids. Clin. Exp. Hypertens. 38, 644–651. 10.1080/10641963.2016.1182182 27669111

[B53] Du W. W. Li X. Li T. Li H. Khorshidi A. Liu F. (2015). The microRNA miR-17-3p inhibits mouse cardiac fibroblast senescence by targeting Par4. J. Cell Sci. 128, 293–304. 10.1242/jcs.158360 25472717

[B54] Edin M. L. Zeldin D. C. (2021). Regulation of cardiovascular biology by microsomal epoxide hydrolase. Toxicol. Res. 37, 285–292. 10.1007/s43188-021-00088-z 34295793 PMC8249505

[B55] El-Sikhry H. E. Alsaleh N. Dakarapu R. Falck J. R. Seubert J. M. (2016). Novel roles of epoxyeicosanoids in regulating cardiac mitochondria. PLoS One 11, e0160380. 10.1371/journal.pone.0160380 27494529 PMC4975494

[B56] Feng T. Meng J. Kou S. Jiang Z. Huang X. Lu Z. (2019). CCN1-Induced cellular senescence promotes heart regeneration. Circulation 139, 2495–2498. 10.1161/CIRCULATIONAHA.119.039530 31107624

[B57] Fu C. Cao Y. Li B. Xu R. Sun Y. Yao Y. (2019). Bradykinin protects cardiac c-kit positive cells from high-glucose-induced senescence through B2 receptor signaling pathway. J. Cell Biochem. 120, 17731–17743. 10.1002/jcb.29039 31119778

[B58] Gan L. Liu D. Liu J. Chen E. Chen C. Liu L. (2021). CD38 deficiency alleviates Ang II-induced vascular remodeling by inhibiting small extracellular vesicle-mediated vascular smooth muscle cell senescence in mice. Signal Transduct. Target Ther. 6, 223. 10.1038/s41392-021-00625-0 34112762 PMC8192533

[B59] Garcia-Prat L. Martinez-Vicente M. Perdiguero E. Ortet L. Rodriguez-Ubreva J. Rebollo E. (2016). Autophagy maintains stemness by preventing senescence. Nature 529, 37–42. 10.1038/nature16187 26738589

[B60] Gardner S. E. Humphry M. Bennett M. R. Clarke M. C. (2015). Senescent vascular smooth muscle cells drive inflammation through an interleukin-1α-dependent senescence-associated secretory phenotype. Arterioscler. Thromb. Vasc. Biol. 35, 1963–1974. 10.1161/ATVBAHA.115.305896 26139463 PMC4548545

[B61] Gary R. K. Kindell S. M. (2005). Quantitative assay of senescence-associated beta-galactosidase activity in mammalian cell extracts. Anal. Biochem. 343, 329–334. 10.1016/j.ab.2005.06.003 16004951

[B62] Gevaert A. B. Shakeri H. Leloup A. J. Van Hove C. E. De Meyer G. R. Y. Vrints C. J. (2017). Endothelial senescence contributes to heart failure with preserved ejection fraction in an aging mouse model. Circ. Heart Fail 10, e003806. 10.1161/CIRCHEARTFAILURE.116.003806 28611124

[B63] Gomes A. P. Price N. L. Ling A. J. Moslehi J. J. Montgomery M. K. Rajman L. (2013). Declining NAD(+) induces a pseudohypoxic state disrupting nuclear-mitochondrial communication during aging. Cell 155, 1624–1638. 10.1016/j.cell.2013.11.037 24360282 PMC4076149

[B64] Goodell S. Cortopassi G. (1998). Analysis of oxygen consumption and mitochondrial permeability with age in mice. Mech. Ageing Dev. 101, 245–256. 10.1016/s0047-6374(97)00182-6 9622228

[B65] Gouspillou G. Sgarioto N. Kapchinsky S. Purves-Smith F. Norris B. Pion C. H. (2014). Increased sensitivity to mitochondrial permeability transition and myonuclear translocation of endonuclease G in atrophied muscle of physically active older humans. FASEB J. 28, 1621–1633. 10.1096/fj.13-242750 24371120

[B66] Grabowska W. Sikora E. Bielak-Zmijewska A. (2017). Sirtuins, a promising target in slowing down the ageing process. Biogerontology 18, 447–476. 10.1007/s10522-017-9685-9 28258519 PMC5514220

[B67] Grazioli S. Pugin J. (2018). Mitochondrial damage-associated molecular patterns: from inflammatory signaling to human diseases. Front. Immunol. 9, 832. 10.3389/fimmu.2018.00832 29780380 PMC5946030

[B68] Griñán-Ferré C. Codony S. Pujol E. Yang J. Leiva R. Escolano C. (2020). Pharmacological inhibition of soluble epoxide hydrolase as a new therapy for Alzheimer’s disease. Neurotherapeutics 17, 1825–1835. 10.1007/s13311-020-00854-1 32488482 PMC7851240

[B69] Grollman A. P. Moriya M. (1993). Mutagenesis by 8-oxoguanine: an enemy within. Trends Genet. 9, 246–249. 10.1016/0168-9525(93)90089-z 8379000

[B70] Grootaert M. O. da Costa Martins P. A. Bitsch N. Pintelon I. De Meyer G. R. Martinet W. (2015). Defective autophagy in vascular smooth muscle cells accelerates senescence and promotes neointima formation and atherogenesis. Autophagy 11, 2014–2032. 10.1080/15548627.2015.1096485 26391655 PMC4824610

[B71] Grootaert M. O. J. Finigan A. Figg N. L. Uryga A. K. Bennett M. R. (2021). SIRT6 protects smooth muscle cells from senescence and reduces atherosclerosis. Circ. Res. 128, 474–491. 10.1161/CIRCRESAHA.120.318353 33353368 PMC7899748

[B72] Gross G. J. Gauthier K. M. Moore J. Falck J. R. Hammock B. D. Campbell W. B. (2008). Effects of the selective EET antagonist, 14,15-EEZE, on cardioprotection produced by exogenous or endogenous EETs in the canine heart. Am. J. Physiol. Heart Circ. Physiol. 294, H2838–H2844. 10.1152/ajpheart.00186.2008 18441205 PMC2863006

[B73] Gu J. Wang S. Guo H. Tan Y. Liang Y. Feng A. (2018). Inhibition of p53 prevents diabetic cardiomyopathy by preventing early-stage apoptosis and cell senescence, reduced glycolysis, and impaired angiogenesis. Cell death and Dis. 9, 82. 10.1038/s41419-017-0093-5 PMC5833384 29362483

[B74] Guo Y. Jia X. Cui Y. Song Y. Wang S. Geng Y. (2021). Sirt3-mediated mitophagy regulates AGEs-induced BMSCs senescence and senile osteoporosis. Redox Biol. 41, 101915. 10.1016/j.redox.2021.101915 33662874 PMC7930642

[B75] Gurr M. I. Harwood J. L. Frayn K. N. (2002). Lipid biochemistry. Springer.

[B76] Hafner A. V. Dai J. Gomes A. P. Xiao C. Y. Palmeira C. M. Rosenzweig A. (2010). Regulation of the mPTP by SIRT3-mediated deacetylation of CypD at lysine 166 suppresses age-related cardiac hypertrophy. Aging (Albany NY). 2, 914–923. 10.18632/aging.100252 21212461 PMC3034180

[B77] Halestrap A. P. (2010). A pore way to die: the role of mitochondria in reperfusion injury and cardioprotection. Biochem. Soc. Trans. 38, 841–860. 10.1042/BST0380841 20658967

[B78] Halestrap A. P. Richardson A. P. (2015). The mitochondrial permeability transition: a current perspective on its identity and role in ischaemia/reperfusion injury. J. Mol. Cell Cardiol. 78, 129–141. 10.1016/j.yjmcc.2014.08.018 25179911

[B79] Handschin C. Spiegelman B. M. (2006). Peroxisome proliferator-activated receptor gamma coactivator 1 coactivators, energy homeostasis, and metabolism. Endocr. Rev. 27, 728–735. 10.1210/er.2006-0037 17018837

[B80] Hayashi T. Matsui-Hirai H. Miyazaki-Akita A. Fukatsu A. Funami J. Ding Q. F. (2006). Endothelial cellular senescence is inhibited by nitric oxide: implications in atherosclerosis associated with menopause and diabetes. Proc. Natl. Acad. Sci. U. S. A. 103, 17018–17023. 10.1073/pnas.0607873103 17075048 PMC1629003

[B81] Hayflick L. Moorhead P. S. (1961). The serial cultivation of human diploid cell strains. Exp. Cell Res. 25, 585–621. 10.1016/0014-4827(61)90192-6 13905658

[B82] He Z. Zhang X. Chen C. Wen Z. Hoopes S. L. Zeldin D. C. (2015). Cardiomyocyte-specific expression of CYP2J2 prevents development of cardiac remodelling induced by angiotensin II. Cardiovasc Res. 105, 304–317. 10.1093/cvr/cvv018 25618409 PMC4351370

[B83] Hernandez-Segura A. de Jong T. V. Melov S. Guryev V. Campisi J. Demaria M. (2017). Unmasking transcriptional heterogeneity in senescent cells. Curr. Biol. 27, 2652–2660. 10.1016/j.cub.2017.07.033 28844647 PMC5788810

[B84] Hong Y. He H. Jiang G. Zhang H. Tao W. Ding Y. (2020). miR-155-5p inhibition rejuvenates aged mesenchymal stem cells and enhances cardioprotection following infarction. Aging Cell 19, e13128. 10.1111/acel.13128 32196916 PMC7189985

[B85] Horn M. A. Trafford A. W. (2016). Aging and the cardiac collagen matrix: novel mediators of fibrotic remodelling. J. Mol. Cell. Cardiol. 93, 175–185. 10.1016/j.yjmcc.2015.11.005 26578393 PMC4945757

[B86] Hoshino A. Mita Y. Okawa Y. Ariyoshi M. Iwai-Kanai E. Ueyama T. (2013). Cytosolic p53 inhibits Parkin-mediated mitophagy and promotes mitochondrial dysfunction in the mouse heart. Nat. Commun. 4, 2308. 10.1038/ncomms3308 23917356

[B87] Hrdlička J. Neckář J. Papoušek F. Husková Z. Kikerlová S. Vaňourková Z. (2019). Epoxyeicosatrienoic acid-based therapy attenuates the progression of postischemic heart failure in normotensive sprague-dawley but not in hypertensive ren-2 transgenic rats. Front. Pharmacol. 10, 159. 10.3389/fphar.2019.00159 30881303 PMC6406051

[B88] Hu C. Zhang X. Teng T. Ma Z. G. Tang Q. Z. (2022). Cellular senescence in cardiovascular diseases: a systematic review. Aging Dis. 13, 103–128. 10.14336/AD.2021.0927 35111365 PMC8782554

[B89] Huang P. Bai L. Liu L. Fu J. Wu K. Liu H. (2021). Redd1 knockdown prevents doxorubicin-induced cardiac senescence. Aging (Albany NY) 13, 13788–13806. 10.18632/aging.202972 33962393 PMC8202877

[B90] Igelmann S. Lessard F. Uchenunu O. Bouchard J. Fernandez-Ruiz A. Rowell M. C. (2021). A hydride transfer complex reprograms NAD metabolism and bypasses senescence. Mol. Cell 81, 3848–3865.e19. 10.1016/j.molcel.2021.08.028 34547241

[B91] Imai S. Guarente L. (2014). NAD+ and sirtuins in aging and disease. Trends Cell Biol. 24, 464–471. 10.1016/j.tcb.2014.04.002 24786309 PMC4112140

[B92] Imig J. D. Cervenka L. Neckar J. (2022). Epoxylipids and soluble epoxide hydrolase in heart diseases. Biochem. Pharmacol. 195, 114866. 10.1016/j.bcp.2021.114866 34863976 PMC8712413

[B93] Imig J. D. Hammock B. D. (2009). Soluble epoxide hydrolase as a therapeutic target for cardiovascular diseases. Nat. Rev. Drug Discov. 8, 794–805. 10.1038/nrd2875 19794443 PMC3021468

[B94] Islam O. Patil P. Goswami S. K. Razdan R. Inamdar M. N. Rizwan M. (2017). Inhibitors of soluble epoxide hydrolase minimize ischemia‐reperfusion‐induced cardiac damage in normal, hypertensive, and diabetic rats. Cardiovasc. Ther. 35, e12259. 10.1111/1755-5922.12259 PMC5597338 28296232

[B95] Ito T. Yagi S. Yamakuchi M. (2010). MicroRNA-34a regulation of endothelial senescence. Biochem. Biophys. Res. Commun. 398, 735–740. 10.1016/j.bbrc.2010.07.012 20627091

[B96] Jamieson K. L. Darwesh A. M. Sosnowski D. K. Zhang H. Shah S. Zhabyeyev P. (2021). Soluble epoxide hydrolase in aged female mice and human explanted hearts following ischemic injury. Int. J. Mol. Sci. 22, 1691. 10.3390/ijms22041691 33567578 PMC7915306

[B97] Jamieson K. L. Endo T. Darwesh A. M. Samokhvalov V. Seubert J. M. (2017a). Cytochrome P450-derived eicosanoids and heart function. Pharmacol. Ther. 179, 47–83. 10.1016/j.pharmthera.2017.05.005 28551025

[B98] Jamieson K. L. Keshavarz-Bahaghighat H. Darwesh A. M. Sosnowski D. K. Seubert J. M. (2020). Age and sex differences in hearts of soluble epoxide hydrolase null mice. Front. Physiol. 11, 48. 10.3389/fphys.2020.00048 32116760 PMC7019103

[B99] Jamieson K. L. Samokhvalov V. Akhnokh M. K. Lee K. Cho W. J. Takawale A. (2017b). Genetic deletion of soluble epoxide hydrolase provides cardioprotective responses following myocardial infarction in aged mice. Prostagl. Other Lipid Mediat 132, 47–58. 10.1016/j.prostaglandins.2017.01.001 28104457

[B100] Jarne-Ferrer J. Griñán-Ferré C. Bellver-Sanchis A. Vázquez S. Muñoz-Torrero D. Pallàs M. (2022). A combined chronic low-dose soluble epoxide hydrolase and acetylcholinesterase pharmacological inhibition promotes memory reinstatement in alzheimer's disease mice models. Pharm. (Basel) 15, 908. 10.3390/ph15080908 PMC9394299 35893732

[B101] Jia L. Zhang W. Ma Y. Chen B. Liu Y. Piao C. (2017). Haplodeficiency of ataxia Telangiectasia mutated accelerates heart failure after myocardial infarction. J. Am. Heart Assoc. 6, e006349. 10.1161/JAHA.117.006349 28724653 PMC5586323

[B102] Jiang X.-s. Xiang X.-y. Chen X.-m. He J.-l. Liu T. Gan H. (2020). Inhibition of soluble epoxide hydrolase attenuates renal tubular mitochondrial dysfunction and ER stress by restoring autophagic flux in diabetic nephropathy. Cell Death and Dis. 11, 385. 10.1038/s41419-020-2594-x PMC7242354 32439839

[B103] Jomova K. Raptova R. Alomar S. Y. Alwasel S. H. Nepovimova E. Kuca K. (2023). Reactive oxygen species, toxicity, oxidative stress, and antioxidants: chronic diseases and aging. Archives Toxicol. 97, 2499–2574. 10.1007/s00204-023-03562-9 PMC10475008 37597078

[B104] Judge S. Leeuwenburgh C. (2007). Cardiac mitochondrial bioenergetics, oxidative stress, and aging. Am. J. Physiol. Cell Physiol. 292, C1983–C1992. 10.1152/ajpcell.00285.2006 17344313

[B105] Kala P. Miklovic M. Jichova S. Skaroupkova P. Vanourkova Z. Maxova H. (2021). Effects of epoxyeicosatrienoic acid-enhancing therapy on the course of congestive heart failure in angiotensin II-dependent rat hypertension: from mRNA analysis towards functional *in vivo* evaluation. Biomedicines 9, 1053. 10.3390/biomedicines9081053 34440257 PMC8393645

[B106] Katragadda D. Batchu S. N. Cho W. J. Chaudhary K. R. Falck J. R. Seubert J. M. (2009). Epoxyeicosatrienoic acids limit damage to mitochondrial function following stress in cardiac cells. J. Mol. Cell Cardiol. 46, 867–875. 10.1016/j.yjmcc.2009.02.028 19285984

[B107] Katsuumi G. Shimizu I. Yoshida Y. Hayashi Y. Ikegami R. Suda M. (2018). Catecholamine-induced senescence of endothelial cells and bone marrow cells promotes cardiac dysfunction in mice. Int. Heart J. 59, 837–844. 10.1536/ihj.17-313 29794381

[B108] Keshavarz-Bahaghighat H. Darwesh A. M. Sosnowski D. K. Seubert J. M. (2020). Mitochondrial dysfunction and inflammaging in heart failure: novel roles of CYP-derived epoxylipids. Cells 9, 1565. 10.3390/cells9071565 32604981 PMC7408578

[B109] Khan S. S. Singer B. D. Vaughan D. E. (2017). Molecular and physiological manifestations and measurement of aging in humans. Aging Cell 16, 624–633. 10.1111/acel.12601 28544158 PMC5506433

[B110] Khavinson V. Linkova N. Dyatlova A. Kantemirova R. Kozlov K. (2022). Senescence-associated secretory phenotype of cardiovascular system cells and inflammaging: perspectives of peptide regulation. Cells 12, 106. 10.3390/cells12010106 36611900 PMC9818427

[B111] Khemais-Benkhiat S. Belcastro E. Idris-Khodja N. Park S. H. Amoura L. Abbas M. (2020). Angiotensin II-induced redox-sensitive SGLT1 and 2 expression promotes high glucose-induced endothelial cell senescence. J. Cell Mol. Med. 24, 2109–2122. 10.1111/jcmm.14233 30929316 PMC7011151

[B112] Kirschner K. Rattanavirotkul N. Quince M. F. Chandra T. (2020). Functional heterogeneity in senescence. Biochem. Soc. Trans. 48, 765–773. 10.1042/BST20190109 32369550 PMC7329341

[B113] Kissova I. Deffieu M. Manon S. Camougrand N. (2004). Uth1p is involved in the autophagic degradation of mitochondria. J. Biol. Chem. 279, 39068–39074. 10.1074/jbc.M406960200 15247238

[B114] Kleele T. Rey T. Winter J. Zaganelli S. Mahecic D. Perreten Lambert H. (2021). Distinct fission signatures predict mitochondrial degradation or biogenesis. Nature 593, 435–439. 10.1038/s41586-021-03510-6 33953403

[B115] Knez J. Winckelmans E. Plusquin M. Thijs L. Cauwenberghs N. Gu Y. (2016). Correlates of peripheral blood mitochondrial DNA content in a general population. Am. J. Epidemiol. 183, 138–146. 10.1093/aje/kwv175 26702630 PMC4706678

[B116] Kompa A. R. Wang B. H. Xu G. Zhang Y. Ho P. Y. Eisennagel S. (2013). Soluble epoxide hydrolase inhibition exerts beneficial anti-remodeling actions post-myocardial infarction. Int. J. Cardiol. 167, 210–219. 10.1016/j.ijcard.2011.12.062 22236509

[B117] Konkel A. Schunck W.-H. (2011). Role of cytochrome P450 enzymes in the bioactivation of polyunsaturated fatty acids. Biochimica Biophysica Acta (BBA)-Proteins Proteomics 1814, 210–222. 10.1016/j.bbapap.2010.09.009 20869469

[B118] Korshunov S. S. Skulachev V. P. Starkov A. A. (1997). High protonic potential actuates a mechanism of production of reactive oxygen species in mitochondria. FEBS Lett. 416, 15–18. 10.1016/s0014-5793(97)01159-9 9369223

[B119] Kosugi R. Shioi T. Watanabe-Maeda K. Yoshida Y. Takahashi K. Machida Y. (2006). Angiotensin II receptor antagonist attenuates expression of aging markers in diabetic mouse heart. Circ. J. 70, 482–488. 10.1253/circj.70.482 16565569

[B120] Kranrod J. W. Darwesh A. M. Bassiouni W. Huang A. Fang L. Korodimas J. V. (2024). Cardioprotective action of a novel synthetic 19,20-EDP analog is sirt dependent. J. Cardiovasc Pharmacol. 83, 105–115. 10.1097/FJC.0000000000001495 38180457 PMC10770468

[B121] Krishna D. R. Sperker B. Fritz P. Klotz U. (1999). Does pH 6 β-galactosidase activity indicate cell senescence? Mech. ageing Dev. 109, 113–123. 10.1016/s0047-6374(99)00031-7 10515661

[B122] Kuilman T. Michaloglou C. Vredeveld L. C. Douma S. van Doorn R. Desmet C. J. (2008). Oncogene-induced senescence relayed by an interleukin-dependent inflammatory network. Cell 133, 1019–1031. 10.1016/j.cell.2008.03.039 18555778

[B123] Kujoth G. C. Hiona A. Pugh T. D. Someya S. Panzer K. Wohlgemuth S. E. (2005). Mitochondrial DNA mutations, oxidative stress, and apoptosis in mammalian aging. Science 309, 481–484. 10.1126/science.1112125 16020738

[B124] Kumari R. Jat P. (2021). Mechanisms of cellular senescence: cell cycle arrest and senescence associated secretory phenotype. Front. Cell Dev. Biol. 9, 645593. 10.3389/fcell.2021.645593 33855023 PMC8039141

[B125] Kurz D. J. Decary S. Hong Y. Erusalimsky J. D. (2000). Senescence-associated (beta)-galactosidase reflects an increase in lysosomal mass during replicative ageing of human endothelial cells. J. Cell Sci. 113 (Pt 20), 3613–3622. 10.1242/jcs.113.20.3613 11017877

[B126] Lakatta E. G. (2015). So! What's aging? Is cardiovascular aging a disease? J. Mol. Cell. Cardiol. 83, 1–13. 10.1016/j.yjmcc.2015.04.005 25870157 PMC4532266

[B127] Lakatta E. G. Levy D. (2003). Arterial and cardiac aging: major shareholders in cardiovascular disease enterprises Part I: aging arteries: a “set up” for vascular disease. Circulation 107, 139–146. 10.1161/01.Cir.0000048892.83521.58 12515756

[B128] La Morgia C. Maresca A. Caporali L. Valentino M. L. Carelli V. (2020). Mitochondrial diseases in adults. J. Intern Med. 287, 592–608. 10.1111/joim.13064 32463135

[B129] Lau L. Porciuncula A. Yu A. Iwakura Y. David G. (2019). Uncoupling the senescence-associated secretory phenotype from cell cycle exit via interleukin-1 inactivation unveils its protumorigenic role. Mol. Cell Biol. 39, e00586–e00518. 10.1128/MCB.00586-18 30988157 PMC6549465

[B130] Lee G. H. Hoang T. H. Jung E. S. Jung S. J. Han S. K. Chung M. J. (2020). Anthocyanins attenuate endothelial dysfunction through regulation of uncoupling of nitric oxide synthase in aged rats. Aging Cell 19, e13279. 10.1111/acel.13279 33274583 PMC7744959

[B131] Lee S. Jeong S. Y. Lim W. C. Kim S. Park Y. Y. Sun X. (2007). Mitochondrial fission and fusion mediators, hFis1 and OPA1, modulate cellular senescence. J. Biol. Chem. 282, 22977–22983. 10.1074/jbc.M700679200 17545159

[B132] Lee S. M. Dho S. H. Ju S. K. Maeng J. S. Kim J. Y. Kwon K. S. (2012). Cytosolic malate dehydrogenase regulates senescence in human fibroblasts. Biogerontology 13, 525–536. 10.1007/s10522-012-9397-0 22971926

[B133] Levraut J. Iwase H. Shao Z. H. Vanden Hoek T. L. Schumacker P. T. (2003). Cell death during ischemia: relationship to mitochondrial depolarization and ROS generation. Am. J. Physiol. Heart Circ. Physiol. 284, H549–H558. 10.1152/ajpheart.00708.2002 12388276

[B134] Li W. Q. Tan S. L. Li X. H. Sun T. L. Li D. Du J. (2019). Calcitonin gene-related peptide inhibits the cardiac fibroblasts senescence in cardiac fibrosis via up-regulating klotho expression. Eur. J. Pharmacol. 843, 96–103. 10.1016/j.ejphar.2018.10.023 30352200

[B135] Li Z. Duan Q. Cui Y. Jones O. D. Shao D. Zhang J. (2023). Cardiac-specific expression of cre recombinase leads to age-related cardiac dysfunction associated with tumor-like growth of atrial cardiomyocyte and ventricular fibrosis and ferroptosis. Int. J. Mol. Sci. 24, 3094. 10.3390/ijms24043094 36834504 PMC9962429

[B136] Liu L. Chen C. Gong W. Li Y. Edin M. L. Zeldin D. C. (2011). Epoxyeicosatrienoic acids attenuate reactive oxygen species level, mitochondrial dysfunction, caspase activation, and apoptosis in carcinoma cells treated with arsenic trioxide. J. Pharmacol. Exp. Ther. 339, 451–463. 10.1124/jpet.111.180505 21846841 PMC3199997

[B137] Liu L. Puri N. Raffaele M. Schragenheim J. Singh S. P. Bradbury J. A. (2018). Ablation of soluble epoxide hydrolase reprogram white fat to beige-like fat through an increase in mitochondrial integrity, HO-1-adiponectin *in vitro* and *in vivo* . Prostagl. Other Lipid Mediat 138, 1–8. 10.1016/j.prostaglandins.2018.07.004 PMC6314013 30041041

[B138] Liu S. Yu C. Xie L. Niu Y. Fu L. (2021). Aerobic exercise improves mitochondrial function in sarcopenia mice through Sestrin2 in an ampkα2-dependent manner. Journals Gerontology Ser. A 76, 1161–1168. 10.1093/gerona/glab029 33512470

[B139] Lopes-Paciencia S. Saint-Germain E. Rowell M. C. Ruiz A. F. Kalegari P. Ferbeyre G. (2019). The senescence-associated secretory phenotype and its regulation. Cytokine 117, 15–22. 10.1016/j.cyto.2019.01.013 30776684

[B140] Lopez-Otin C. Blasco M. A. Partridge L. Serrano M. Kroemer G. (2013). The hallmarks of aging. Cell 153, 1194–1217. 10.1016/j.cell.2013.05.039 23746838 PMC3836174

[B141] Lopez-Otin C. Blasco M. A. Partridge L. Serrano M. Kroemer G. (2023). Hallmarks of aging: an expanding universe. Cell 186, 243–278. 10.1016/j.cell.2022.11.001 36599349

[B142] Lyu G. Guan Y. Zhang C. Zong L. Sun L. Huang X. (2018). TGF-β signaling alters H4K20me3 status via miR-29 and contributes to cellular senescence and cardiac aging. Nat. Commun. 9, 2560. 10.1038/s41467-018-04994-z 29967491 PMC6028646

[B143] Ma Y. Zheng B. Zhang X. H. Nie Z. Y. Yu J. Zhang H. (2022). Correction for: circACTA2 mediates Ang II-induced VSMC senescence by modulation of the interaction of ILF3 with CDK4 mRNA. Aging (Albany NY) 14, 7186–7188. 10.18632/aging.204274 36096998 PMC9512494

[B144] Mai S. Klinkenberg M. Auburger G. Bereiter-Hahn J. Jendrach M. (2010). Decreased expression of Drp1 and Fis1 mediates mitochondrial elongation in senescent cells and enhances resistance to oxidative stress through PINK1. J. Cell Sci. 123, 917–926. 10.1242/jcs.059246 20179104

[B145] Man A. W. C. Li H. Xia N. (2019). The role of Sirtuin1 in regulating endothelial function, arterial remodeling and vascular aging. Front. Physiol. 10, 1173. 10.3389/fphys.2019.01173 31572218 PMC6751260

[B146] Mather M. Rottenberg H. (2000). Aging enhances the activation of the permeability transition pore in mitochondria. Biochem. Biophys. Res. Commun. 273, 603–608. 10.1006/bbrc.2000.2994 10873652

[B147] Matthews C. Gorenne I. Scott S. Figg N. Kirkpatrick P. Ritchie A. (2006). Vascular smooth muscle cells undergo telomere-based senescence in human atherosclerosis: effects of telomerase and oxidative stress. Circ. Res. 99, 156–164. 10.1161/01.RES.0000233315.38086.bc 16794190

[B148] Mengel-From J. Thinggaard M. Dalgård C. Kyvik K. O. Christensen K. Christiansen L. (2014). Mitochondrial DNA copy number in peripheral blood cells declines with age and is associated with general health among elderly. Hum. Genet. 133, 1149–1159. 10.1007/s00439-014-1458-9 24902542 PMC4127366

[B149] Merabet N. Bellien J. Glevarec E. Nicol L. Lucas D. Remy-Jouet I. (2012). Soluble epoxide hydrolase inhibition improves myocardial perfusion and function in experimental heart failure. J. Mol. Cell Cardiol. 52, 660–666. 10.1016/j.yjmcc.2011.11.015 22155238

[B150] Meyer K. Hodwin B. Ramanujam D. Engelhardt S. Sarikas A. (2016). Essential role for premature senescence of myofibroblasts in myocardial fibrosis. J. Am. Coll. Cardiol. 67, 2018–2028. 10.1016/j.jacc.2016.02.047 27126529

[B151] Miao S. B. Xie X. L. Yin Y. J. Zhao L. L. Zhang F. Shu Y. N. (2017). Accumulation of smooth muscle 22α protein accelerates senescence of vascular smooth muscle cells via stabilization of p53 *in vitro* and *in vivo* . Arterioscler. Thromb. Vasc. Biol. 37, 1849–1859. 10.1161/ATVBAHA.117.309378 28798142

[B152] Mijit M. Caracciolo V. Melillo A. Amicarelli F. Giordano A. (2020). Role of p53 in the regulation of cellular senescence. Biomolecules 10, 420. 10.3390/biom10030420 32182711 PMC7175209

[B153] Min L. J. Mogi M. Iwanami J. Li J. M. Sakata A. Fujita T. (2007). Cross-talk between aldosterone and angiotensin II in vascular smooth muscle cell senescence. Cardiovasc Res. 76, 506–516. 10.1016/j.cardiores.2007.07.008 17706954

[B154] Minamino T. Miyauchi H. Yoshida T. Ishida Y. Yoshida H. Komuro I. (2002). Endothelial cell senescence in human atherosclerosis: role of telomere in endothelial dysfunction. Circulation 105, 1541–1544. 10.1161/01.cir.0000013836.85741.17 11927518

[B155] Minamino T. Yoshida T. Tateno K. Miyauchi H. Zou Y. Toko H. (2003). Ras induces vascular smooth muscle cell senescence and inflammation in human atherosclerosis. Circulation 108, 2264–2269. 10.1161/01.CIR.0000093274.82929.22 14557365

[B156] Mitry M. A. Laurent D. Keith B. L. Sira E. Eisenberg C. A. Eisenberg L. M. (2020). Accelerated cardiomyocyte senescence contributes to late-onset doxorubicin-induced cardiotoxicity. Am. J. Physiol. Cell Physiol. 318, C380–C391. 10.1152/ajpcell.00073.2019 31913702 PMC7052608

[B157] Miwa S. Jow H. Baty K. Johnson A. Czapiewski R. Saretzki G. (2014). Low abundance of the matrix arm of complex I in mitochondria predicts longevity in mice. Nat. Commun. 5, 3837. 10.1038/ncomms4837 24815183 PMC4024759

[B158] Miwa S. Kashyap S. Chini E. von Zglinicki T. (2022). Mitochondrial dysfunction in cell senescence and aging. J. Clin. investigation 132, e158447. 10.1172/JCI158447 PMC9246372 35775483

[B159] Monti J. Fischer J. Paskas S. Heinig M. Schulz H. Gösele C. (2008). Soluble epoxide hydrolase is a susceptibility factor for heart failure in a rat model of human disease. Nat. Genet. 40, 529–537. 10.1038/ng.129 18443590 PMC7370537

[B160] Monzel A. S. Enriquez J. A. Picard M. (2023). Multifaceted mitochondria: moving mitochondrial science beyond function and dysfunction. Nat. Metab. 5, 546–562. 10.1038/s42255-023-00783-1 37100996 PMC10427836

[B161] Moskalev A. (2019). Biomarkers of human aging. Cham: Springer International Publishing, 1–4.

[B162] Moslehi J. DePinho R. A. Sahin E. (2012). Telomeres and mitochondria in the aging heart. Circulation Res. 110, 1226–1237. 10.1161/CIRCRESAHA.111.246868 22539756 PMC3718635

[B163] Mouchiroud L. Houtkooper R. H. Moullan N. Katsyuba E. Ryu D. Cantó C. (2013). The NAD+/sirtuin pathway modulates longevity through activation of mitochondrial UPR and FOXO signaling. Cell 154, 430–441. 10.1016/j.cell.2013.06.016 23870130 PMC3753670

[B164] Nacarelli T. Azar A. Sell C. (2016). Mitochondrial stress induces cellular senescence in an mTORC1-dependent manner. Free Radic. Biol. Med. 95, 133–154. 10.1016/j.freeradbiomed.2016.03.008 27016071

[B165] Nakahira K. Hisata S. Choi A. M. (2015). The roles of mitochondrial damage-associated molecular patterns in diseases. Antioxid. Redox Signal 23, 1329–1350. 10.1089/ars.2015.6407 26067258 PMC4685486

[B166] Nakamura T. Hosoyama T. Murakami J. Samura M. Ueno K. Kurazumi H. (2017). Age-related increase in Wnt inhibitor causes a senescence-like phenotype in human cardiac stem cells. Biochem. Biophys. Res. Commun. 487, 653–659. 10.1016/j.bbrc.2017.04.110 28435069

[B167] Naqvi N. Li M. Calvert J. W. Tejada T. Lambert J. P. Wu J. (2014). A proliferative burst during preadolescence establishes the final cardiomyocyte number. Cell 157, 795–807. 10.1016/j.cell.2014.03.035 24813607 PMC4078902

[B168] Neckar J. Hye Khan M. A. Gross G. J. Cyprova M. Hrdlicka J. Kvasilova A. (2019). Epoxyeicosatrienoic acid analog EET-B attenuates post-myocardial infarction remodeling in spontaneously hypertensive rats. Clin. Sci. (Lond). 133, 939–951. 10.1042/CS20180728 30979784 PMC6492034

[B169] Neckář J. Kopkan L. Husková Z. Kolář F. Papoušek F. Kramer H. J. (2012). Inhibition of soluble epoxide hydrolase by cis-4-[4-(3-adamantan-1-ylureido) cyclohexyl-oxy] benzoic acid exhibits antihypertensive and cardioprotective actions in transgenic rats with angiotensin II-dependent hypertension. Clin. Sci. 122, 513–525. 10.1042/CS20110622 PMC3528350 22324471

[B170] Nelson G. Kucheryavenko O. Wordsworth J. von Zglinicki T. (2018). The senescent bystander effect is caused by ROS-activated NF-κB signalling. Mech. Ageing Dev. 170, 30–36. 10.1016/j.mad.2017.08.005 28837845 PMC5861994

[B171] Nelson J. W. Young J. M. Borkar R. N. Woltjer R. L. Quinn J. F. Silbert L. C. (2014). Role of soluble epoxide hydrolase in age-related vascular cognitive decline. Prostagl. Other Lipid Mediat 113-115, 30–37. 10.1016/j.prostaglandins.2014.09.003 PMC4254026 25277097

[B172] Nie S. Lu J. Wang L. Gao M. (2020). Pro‐inflammatory role of cell‐free mitochondrial DNA in cardiovascular diseases. IUBMB life 72, 1879–1890. 10.1002/iub.2339 32656943

[B173] Ock S. Lee W. S. Ahn J. Kim H. M. Kang H. Kim H. S. (2016). Deletion of IGF-1 receptors in cardiomyocytes attenuates cardiac aging in male mice. Endocrinology 157, 336–345. 10.1210/en.2015-1709 26469138 PMC4701888

[B174] O’Leary M. F. Vainshtein A. Iqbal S. Ostojic O. Hood D. A. (2013). Adaptive plasticity of autophagic proteins to denervation in aging skeletal muscle. Am. J. Physiol. Cell Physiol. 304, C422–C430. 10.1152/ajpcell.00240.2012 23220115

[B175] Oni-Orisan A. Alsaleh N. Lee C. R. Seubert J. M. (2014). Epoxyeicosatrienoic acids and cardioprotection: the road to translation. J. Mol. Cell Cardiol. 74, 199–208. 10.1016/j.yjmcc.2014.05.016 24893205 PMC4115045

[B176] Ota H. Akishita M. Eto M. Iijima K. Kaneki M. Ouchi Y. (2007). Sirt1 modulates premature senescence-like phenotype in human endothelial cells. J. Mol. Cell Cardiol. 43, 571–579. 10.1016/j.yjmcc.2007.08.008 17916362

[B177] Pagan L. U. Gomes M. J. Gatto M. Mota G. A. F. Okoshi K. Okoshi M. P. (2022). The role of oxidative stress in the aging heart. Antioxidants (Basel) 11, 336. 10.3390/antiox11020336 35204217 PMC8868312

[B178] Pagliuso A. Cossart P. Stavru F. (2018). The ever-growing complexity of the mitochondrial fission machinery. Cell Mol. Life Sci. 75, 355–374. 10.1007/s00018-017-2603-0 28779209 PMC5765209

[B179] Parodi-Rullán R. M. Chapa-Dubocq X. R. Javadov S. (2018). Acetylation of mitochondrial proteins in the heart: the role of SIRT3. Front. Physiol. 9, 1094. 10.3389/fphys.2018.01094 30131726 PMC6090200

[B180] Passos J. F. Nelson G. Wang C. Richter T. Simillion C. Proctor C. J. (2010). Feedback between p21 and reactive oxygen production is necessary for cell senescence. Mol. Syst. Biol. 6, 347. 10.1038/msb.2010.5 20160708 PMC2835567

[B181] Passos J. F. Saretzki G. Ahmed S. Nelson G. Richter T. Peters H. (2007). Mitochondrial dysfunction accounts for the stochastic heterogeneity in telomere-dependent senescence. PLoS Biol. 5, e110. 10.1371/journal.pbio.0050110 17472436 PMC1858712

[B182] Pastorino J. G. Tafani M. Rothman R. J. Marcinkeviciute A. Hoek J. B. Farber J. L. (1999). Functional consequences of the sustained or transient activation by Bax of the mitochondrial permeability transition pore. J. Biol. Chem. 274, 31734–31739. 10.1074/jbc.274.44.31734 10531385

[B183] Picca A. Lezza A. M. (2015). Regulation of mitochondrial biogenesis through TFAM-mitochondrial DNA interactions: useful insights from aging and calorie restriction studies. Mitochondrion 25, 67–75. 10.1016/j.mito.2015.10.001 26437364

[B184] Pignolo R. J. Passos J. F. Khosla S. Tchkonia T. Kirkland J. L. (2020). Reducing senescent cell burden in aging and disease. Trends Mol. Med. 26, 630–638. 10.1016/j.molmed.2020.03.005 32589933 PMC7857028

[B185] Pillai V. B. Samant S. Hund S. Gupta M. Gupta M. P. (2021). The nuclear sirtuin SIRT6 protects the heart from developing aging-associated myocyte senescence and cardiac hypertrophy. Aging (Albany NY) 13, 12334–12358. 10.18632/aging.203027 33934090 PMC8148452

[B186] Pinti M. Cevenini E. Nasi M. De Biasi S. Salvioli S. Monti D. (2014). Circulating mitochondrial DNA increases with age and is a familiar trait: implications for “inflamm-aging”. Eur. J. Immunol. 44, 1552–1562. 10.1002/eji.201343921 24470107

[B187] Porrello E. R. Mahmoud A. I. Simpson E. Hill J. A. Richardson J. A. Olson E. N. (2011). Transient regenerative potential of the neonatal mouse heart. Science 331, 1078–1080. 10.1126/science.1200708 21350179 PMC3099478

[B188] Qiu H. Li N. Liu J. Y. Harris T. R. Hammock B. D. Chiamvimonvat N. (2011). Soluble epoxide hydrolase inhibitors and heart failure. Cardiovasc. Ther. 29, 99–111. 10.1111/j.1755-5922.2010.00150.x 20433684 PMC3325372

[B189] Qu Y. Cao J. Wang D. Wang S. Li Y. Zhu Y. (2022). 14,15-Epoxyeicosatrienoic acid protect against glucose deprivation and reperfusion-induced cerebral microvascular endothelial cells injury by modulating mitochondrial autophagy via SIRT1/FOXO3a signaling pathway and TSPO protein. Front. Cell Neurosci. 16, 888836. 10.3389/fncel.2022.888836 35558879 PMC9086968

[B190] Rana A. Oliveira M. P. Khamoui A. V. Aparicio R. Rera M. Rossiter H. B. (2017). Promoting Drp1-mediated mitochondrial fission in midlife prolongs healthy lifespan of *Drosophila melanogaster* . Nat. Commun. 8, 448. 10.1038/s41467-017-00525-4 28878259 PMC5587646

[B191] Rebelo A. P. Dillon L. M. Moraes C. T. (2011). Mitochondrial DNA transcription regulation and nucleoid organization. J. Inherit. Metab. Dis. 34, 941–951. 10.1007/s10545-011-9330-8 21541724

[B192] Riley J. S. Tait S. W. (2020). Mitochondrial DNA in inflammation and immunity. EMBO Rep. 21, e49799. 10.15252/embr.201949799 32202065 PMC7132203

[B193] Rizza S. Cardaci S. Montagna C. Di Giacomo G. De Zio D. Bordi M. (2018). S-nitrosylation drives cell senescence and aging in mammals by controlling mitochondrial dynamics and mitophagy. Proc. Natl. Acad. Sci. 115, E3388–E3397. 10.1073/pnas.1722452115 29581312 PMC5899480

[B194] Robbins E. Levine E. M. Eagle H. (1970). Morphologic changes accompanying senescence of cultured human diploid cells. J. Exp. Med. 131, 1211–1222. 10.1084/jem.131.6.1211 5419270 PMC2138843

[B195] Rodier F. Coppe J. P. Patil C. K. Hoeijmakers W. A. Munoz D. P. Raza S. R. (2009). Persistent DNA damage signalling triggers senescence-associated inflammatory cytokine secretion. Nat. Cell Biol. 11, 973–979. 10.1038/ncb1909 19597488 PMC2743561

[B196] Samokhvalov V. Alsaleh N. El-Sikhry H. E. Jamieson K. L. Chen C. B. Lopaschuk D. G. (2013). Epoxyeicosatrienoic acids protect cardiac cells during starvation by modulating an autophagic response. Cell Death Dis. 4, e885. 10.1038/cddis.2013.418 24157879 PMC3920965

[B197] Samokhvalov V. Jamieson K. L. Darwesh A. M. Keshavarz-Bahaghighat H. Lee T. Y. T. Edin M. (2018). Deficiency of soluble epoxide hydrolase protects cardiac function impaired by LPS-induced acute inflammation. Front. Pharmacol. 9, 1572. 10.3389/fphar.2018.01572 30692927 PMC6339940

[B198] Sarig R. Rimmer R. Bassat E. Zhang L. Umansky K. B. Lendengolts D. (2019). Transient p53-mediated regenerative senescence in the injured heart. Circulation 139, 2491–2494. 10.1161/CIRCULATIONAHA.119.040125 31107623

[B199] Sarkar P. Zaja I. Bienengraeber M. Rarick K. R. Terashvili M. Canfield S. (2014). Epoxyeicosatrienoic acids pretreatment improves amyloid β-induced mitochondrial dysfunction in cultured rat hippocampal astrocytes. Am. J. Physiol. Heart Circ. Physiol. 306, H475–H484. 10.1152/ajpheart.00001.2013 24285116 PMC3920242

[B200] Sawaki D. Czibik G. Pini M. Ternacle J. Suffee N. Mercedes R. (2018). Visceral adipose tissue drives cardiac aging through modulation of fibroblast senescence by osteopontin production. Circulation 138, 809–822. 10.1161/circulationaha.117.031358 29500246

[B201] Sawyer D. B. Colucci W. S. (2000). Mitochondrial oxidative stress in heart failure: “oxygen wastage” revisited. Circulation Res. 86, 119–120. 10.1161/01.res.86.2.119 10666404

[B202] Scarpulla R. C. (2011). Metabolic control of mitochondrial biogenesis through the PGC-1 family regulatory network. Biochimica biophysica acta (BBA)-molecular Cell Res. 1813, 1269–1278. 10.1016/j.bbamcr.2010.09.019 PMC3035754 20933024

[B203] Schunck W. H. Konkel A. Fischer R. Weylandt K. H. (2018). Therapeutic potential of omega-3 fatty acid-derived epoxyeicosanoids in cardiovascular and inflammatory diseases. Pharmacol. Ther. 183, 177–204. 10.1016/j.pharmthera.2017.10.016 29080699

[B204] Seabright A. P. Fine N. H. F. Barlow J. P. Lord S. O. Musa I. Gray A. (2020). AMPK activation induces mitophagy and promotes mitochondrial fission while activating TBK1 in a PINK1-Parkin independent manner. Faseb J. 34, 6284–6301. 10.1096/fj.201903051R 32201986 PMC7212019

[B205] Sebastián D. Sorianello E. Segalés J. Irazoki A. Ruiz-Bonilla V. Sala D. (2016). Mfn2 deficiency links age-related sarcopenia and impaired autophagy to activation of an adaptive mitophagy pathway. Embo J. 35, 1677–1693. 10.15252/embj.201593084 27334614 PMC4969577

[B206] Sharifi-Sanjani M. Oyster N. M. Tichy E. D. Bedi K. C. Jr. Harel O. Margulies K. B. (2017). Cardiomyocyte-specific telomere shortening is a distinct signature of heart failure in humans. J. Am. Heart Assoc. 6, e005086. 10.1161/JAHA.116.005086 28882819 PMC5634248

[B207] Sharpless N. E. Sherr C. J. (2015). Forging a signature of *in vivo* senescence. Nat. Rev. Cancer 15, 397–408. 10.1038/nrc3960 26105537

[B208] Shi T. van Soest D. M. Polderman P. E. Burgering B. M. Dansen T. B. (2021). DNA damage and oxidant stress activate p53 through differential upstream signaling pathways. Free Radic. Biol. Med. 172, 298–311. 10.1016/j.freeradbiomed.2021.06.013 34144191

[B209] Shibamoto M. Higo T. Naito A. T. Nakagawa A. Sumida T. Okada K. (2019). Activation of DNA damage response and cellular senescence in cardiac fibroblasts limit cardiac fibrosis after myocardial infarction. Int. Heart J. 60, 944–957. 10.1536/ihj.18-701 31257341

[B210] Shimada K. Crother T. R. Karlin J. Dagvadorj J. Chiba N. Chen S. (2012). Oxidized mitochondrial DNA activates the NLRP3 inflammasome during apoptosis. Immunity 36, 401–414. 10.1016/j.immuni.2012.01.009 22342844 PMC3312986

[B211] Shimizu I. Minamino T. (2019). Cellular senescence in cardiac diseases. J. Cardiol. 74, 313–319. 10.1016/j.jjcc.2019.05.002 31202488

[B212] Sirish P. Li N. Liu J.-Y. Lee K. S. S. Hwang S. H. Qiu H. (2013). Unique mechanistic insights into the beneficial effects of soluble epoxide hydrolase inhibitors in the prevention of cardiac fibrosis. Proc. Natl. Acad. Sci. 110, 5618–5623. 10.1073/pnas.1221972110 23493561 PMC3619365

[B213] Sirish P. Li N. Timofeyev V. Zhang X. D. Wang L. Yang J. (2016). Molecular mechanisms and new treatment paradigm for atrial fibrillation. Circ. Arrhythm. Electrophysiol. 9. 10.1161/CIRCEP.115.003721 PMC4869994 27162031

[B214] Sitte N. Merker K. Grune T. von Zglinicki T. (2001). Lipofuscin accumulation in proliferating fibroblasts *in vitro*: an indicator of oxidative stress. Exp. Gerontol. 36, 475–486. 10.1016/s0531-5565(00)00253-9 11250119

[B215] Smith W. L. Murphy R. C. (2016). “The eicosanoids: cyclooxygenase, lipoxygenase and epoxygenase pathways,” in Biochemistry of lipids, lipoproteins and membranes (Elsevier), 259–296.

[B216] Sokoła-Wysoczańska E. Wysoczański T. Wagner J. Czyż K. Bodkowski R. Lochyński S. (2018). Polyunsaturated fatty acids and their potential therapeutic role in cardiovascular system disorders—a review. Nutrients 10, 1561. 10.3390/nu10101561 30347877 PMC6213446

[B217] Sokolova M. Vinge L. E. Alfsnes K. Olsen M. B. Eide L. Kaasboll O. J. (2017). Palmitate promotes inflammatory responses and cellular senescence in cardiac fibroblasts. Biochim. Biophys. Acta Mol. Cell Biol. Lipids 1862, 234–245. 10.1016/j.bbalip.2016.11.003 27845246

[B218] Song H. Conte J. V. Jr. Foster A. H. McLaughlin J. S. Wei C. (1999). Increased p53 protein expression in human failing myocardium. J. Heart Lung Transpl. 18, 744–749. 10.1016/s1053-2498(98)00039-4 10512520

[B219] Song Y. Shen H. Schenten D. Shan P. Lee P. J. Goldstein D. R. (2012). Aging enhances the basal production of IL-6 and CCL2 in vascular smooth muscle cells. Arterioscler. Thromb. Vasc. Biol. 32, 103–109. 10.1161/ATVBAHA.111.236349 22034510 PMC3241880

[B220] Sosnowski D. K. Jamieson K. L. Darwesh A. M. Zhang H. Keshavarz-Bahaghighat H. Valencia R. (2022a). Changes in the left ventricular eicosanoid profile in human dilated cardiomyopathy. Front. Cardiovasc Med. 9, 879209. 10.3389/fcvm.2022.879209 35665247 PMC9160304

[B221] Sosnowski D. K. Jamieson K. L. Gruzdev A. Li Y. Valencia R. Yousef A. (2022b). Cardiomyocyte-specific disruption of soluble epoxide hydrolase limits inflammation to preserve cardiac function. Am. J. Physiol. Heart Circ. Physiol. 323, H670–H687. 10.1152/ajpheart.00217.2022 35985007 PMC9512117

[B222] Srivastava S. (2017). The mitochondrial basis of aging and age-related disorders. Genes 8, 398. 10.3390/genes8120398 29257072 PMC5748716

[B223] Stevenson M. D. Canugovi C. Vendrov A. E. Hayami T. Bowles D. E. Krause K. H. (2019). NADPH oxidase 4 regulates inflammation in ischemic heart failure: role of soluble epoxide hydrolase. Antioxid. Redox Signal 31, 39–58. 10.1089/ars.2018.7548 30450923 PMC6552006

[B224] Sudhahar V. Shaw S. Imig J. D. (2010). Epoxyeicosatrienoic acid analogs and vascular function. Curr. Med. Chem. 17, 1181–1190. 10.2174/092986710790827843 20158473 PMC2855336

[B225] Sun C. Simon S. I. Foster G. A. Radecke C. E. Hwang H. V. Zhang X. (2016). 11,12-Epoxyecosatrienoic acids mitigate endothelial dysfunction associated with estrogen loss and aging: role of membrane depolarization. J. Mol. Cell Cardiol. 94, 180–188. 10.1016/j.yjmcc.2016.03.019 27079253 PMC4972711

[B226] Sun W. Liu C. Chen Q. Liu N. Yan Y. Liu B. (2018). SIRT3: a new regulator of cardiovascular diseases. Oxid. Med. Cell Longev. 2018, 7293861. 10.1155/2018/7293861 29643974 PMC5831850

[B227] Takubo K. Izumiyama-Shimomura N. Honma N. Sawabe M. Arai T. Kato M. (2002). Telomere lengths are characteristic in each human individual. Exp. Gerontol. 37, 523–531. 10.1016/s0531-5565(01)00218-2 11830355

[B228] Tan P. Guo Y. H. Zhan J. K. Long L. M. Xu M. L. Ye L. (2019). LncRNA-ANRIL inhibits cell senescence of vascular smooth muscle cells by regulating miR-181a/Sirt1. Biochem. Cell Biol. 97, 571–580. 10.1139/bcb-2018-0126 30789795

[B229] Tang X. Li P. H. Chen H. Z. (2020). Cardiomyocyte senescence and cellular communications within myocardial microenvironments. Front. Endocrinol. (Lausanne) 11, 280. 10.3389/fendo.2020.00280 32508749 PMC7253644

[B230] Theken K. N. Schuck R. N. Edin M. L. Tran B. Ellis K. Bass A. (2012). Evaluation of cytochrome P450-derived eicosanoids in humans with stable atherosclerotic cardiovascular disease. Atherosclerosis 222, 530–536. 10.1016/j.atherosclerosis.2012.03.022 22503544 PMC3361525

[B231] Toko H. Hariharan N. Konstandin M. H. Ormachea L. McGregor M. Gude N. A. (2014). Differential regulation of cellular senescence and differentiation by prolyl isomerase Pin1 in cardiac progenitor cells. J. Biol. Chem. 289, 5348–5356. 10.1074/jbc.M113.526442 24375406 PMC3937613

[B232] Topcu A. Kostakoglu U. Mercantepe T. Yilmaz H. K. Tumkaya L. Uydu H. A. (2022). The cardioprotective effects of perindopril in a model of polymicrobial sepsis: the role of radical oxygen species and the inflammation pathway. J. Biochem. Mol. Toxicol. 36, e23080. 10.1002/jbt.23080 35417068

[B233] Trifunovic A. Hansson A. Wredenberg A. Rovio A. T. Dufour E. Khvorostov I. (2005). Somatic mtDNA mutations cause aging phenotypes without affecting reactive oxygen species production. Proc. Natl. Acad. Sci. 102, 17993–17998. 10.1073/pnas.0508886102 16332961 PMC1312403

[B234] Trifunovic A. Larsson N. G. (2008). Mitochondrial dysfunction as a cause of ageing. J. Intern. Med. 263, 167–178. 10.1111/j.1365-2796.2007.01905.x 18226094

[B235] Triposkiadis F. Butler J. Abboud F. M. Armstrong P. W. Adamopoulos S. Atherton J. J. (2019a). The continuous heart failure spectrum: moving beyond an ejection fraction classification. Eur. heart J. 40, 2155–2163. 10.1093/eurheartj/ehz158 30957868 PMC7963129

[B236] Triposkiadis F. Xanthopoulos A. Butler J. (2019b). Cardiovascular aging and heart failure: JACC review topic of the week. J. Am. Coll. Cardiol. 74, 804–813. 10.1016/j.jacc.2019.06.053 31395131

[B237] Tsai I. C. Pan Z. C. Cheng H. P. Liu C. H. Lin B. T. Jiang M. J. (2016). Reactive oxygen species derived from NADPH oxidase 1 and mitochondria mediate angiotensin II-induced smooth muscle cell senescence. J. Mol. Cell Cardiol. 98, 18–27. 10.1016/j.yjmcc.2016.07.001 27381955

[B238] Twig G. Shirihai O. S. (2011). The interplay between mitochondrial dynamics and mitophagy. Antioxid. Redox Signal 14, 1939–1951. 10.1089/ars.2010.3779 21128700 PMC3078508

[B239] Valencia R. Bassiouni W. Darwesh A. M. Bapuji R. Seubert J. M. (2022). Cardiomyocyte-specific CYP2J2 and its therapeutic implications. Expert Opin. Drug Metab. Toxicol. 18, 423–439. 10.1080/17425255.2022.2114344 35997132

[B240] ValenciaR.KranrodJ. W.FangL.SolimanA. M.AzerB.Clemente-CasaresX. (2024). Linoleic acid-derived diol 12,13-DiHOME enhances NLRP3 inflammasome activation in macrophages. FASEB J. 38, e23748. 10.1096/fj.202301640RR 38940767

[B241] Vassallo P. F. Simoncini S. Ligi I. Chateau A. L. Bachelier R. Robert S. (2014). Accelerated senescence of cord blood endothelial progenitor cells in premature neonates is driven by SIRT1 decreased expression. Blood 123, 2116–2126. 10.1182/blood-2013-02-484956 24518759

[B242] Velarde M. C. Flynn J. M. Day N. U. Melov S. Campisi J. (2012). Mitochondrial oxidative stress caused by Sod2 deficiency promotes cellular senescence and aging phenotypes in the skin. Aging (Albany NY) 4, 3–12. 10.18632/aging.100423 22278880 PMC3292901

[B243] Victorelli S. Salmonowicz H. Chapman J. Martini H. Vizioli M. G. Riley J. S. (2023). Apoptotic stress causes mtDNA release during senescence and drives the SASP. Nature 622, 627–636. 10.1038/s41586-023-06621-4 37821702 PMC10584674

[B244] Voghel G. Thorin-Trescases N. Farhat N. Nguyen A. Villeneuve L. Mamarbachi A. M. (2007). Cellular senescence in endothelial cells from atherosclerotic patients is accelerated by oxidative stress associated with cardiovascular risk factors. Mech. Ageing Dev. 128, 662–671. 10.1016/j.mad.2007.09.006 18022214

[B245] Waldman M. Bellner L. Vanella L. Schragenheim J. Sodhi K. Singh S. P. (2016). Epoxyeicosatrienoic acids regulate adipocyte differentiation of mouse 3T3 cells, via PGC-1α activation, which is required for HO-1 expression and increased mitochondrial function. Stem Cells Dev. 25, 1084–1094. 10.1089/scd.2016.0072 27224420 PMC4939374

[B246] Wang J. Uryga A. K. Reinhold J. Figg N. Baker L. Finigan A. (2015a). Vascular smooth muscle cell senescence promotes atherosclerosis and features of plaque vulnerability. Circulation 132, 1909–1919. 10.1161/CIRCULATIONAHA.115.016457 26416809

[B247] Wang K. Du Y. Li P. Guan C. Zhou M. Wu L. (2024). Nanoplastics causes heart aging/myocardial cell senescence through the Ca(2+)/mtDNA/cGAS-STING signaling cascade. J. Nanobiotechnology 22, 96. 10.1186/s12951-024-02375-x 38448951 PMC10918962

[B248] Wang L. Chen M. Yuan L. Xiang Y. Zheng R. Zhu S. (2014). 14,15-EET promotes mitochondrial biogenesis and protects cortical neurons against oxygen/glucose deprivation-induced apoptosis. Biochem. Biophys. Res. Commun. 450, 604–609. 10.1016/j.bbrc.2014.06.022 24931672

[B249] Wang W. Wagner K. M. Wang Y. Singh N. Yang J. He Q. (2023). Soluble epoxide hydrolase contributes to cell senescence and ER stress in aging mice colon. Int. J. Mol. Sci. 24, 4570. 10.3390/ijms24054570 36901999 PMC10003560

[B250] Wang X. Guo Z. Ding Z. Khaidakov M. Lin J. Xu Z. (2015b). Endothelin-1 upregulation mediates aging-related cardiac fibrosis. J. Mol. Cell Cardiol. 80, 101–109. 10.1016/j.yjmcc.2015.01.001 25584774

[B251] Wang Y. C. Lee A. S. Lu L. S. Ke L. Y. Chen W. Y. Dong J. W. (2018). Human electronegative LDL induces mitochondrial dysfunction and premature senescence of vascular cells *in vivo* . Aging Cell 17, e12792. 10.1111/acel.12792 29923368 PMC6052487

[B252] Watroba M. Szukiewicz D. (2016). The role of sirtuins in aging and age-related diseases. Adv. Med. Sci. 61, 52–62. 10.1016/j.advms.2015.09.003 26521204

[B253] Wei S. Xiao Z. Huang J. Peng Z. Zhang B. Li W. (2022). Disulfiram inhibits oxidative stress and NLRP3 inflammasome activation to prevent LPS-induced cardiac injury. Int. Immunopharmacol. 105, 108545. 10.1016/j.intimp.2022.108545 35091339

[B254] Westphal C. Konkel A. Schunck W. H. (2015). Cytochrome p450 enzymes in the bioactivation of polyunsaturated Fatty acids and their role in cardiovascular disease. Adv. Exp. Med. Biol. 851, 151–187. 10.1007/978-3-319-16009-2_6 26002735

[B255] Westphal C. Spallek B. Konkel A. Marko L. Qadri F. DeGraff L. M. (2013). CYP2J2 overexpression protects against arrhythmia susceptibility in cardiac hypertrophy. PLoS One 8, e73490. 10.1371/journal.pone.0073490 24023684 PMC3758319

[B256] Widjaja A. A. Lim W.-W. Viswanathan S. Chothani S. Corden B. Dasan C. M. (2024). Inhibition of IL-11 signalling extends mammalian healthspan and lifespan. Nature 1-9. 10.1038/s41586-024-07701-9 PMC11291288 39020175

[B257] Wiley C. D. Velarde M. C. Lecot P. Liu S. Sarnoski E. A. Freund A. (2016). Mitochondrial dysfunction induces senescence with a distinct secretory phenotype. Cell Metab. 23, 303–314. 10.1016/j.cmet.2015.11.011 26686024 PMC4749409

[B258] Wolf A. M. (2021). MtDNA mutations and aging-not a closed case after all? Signal Transduct. Target Ther. 6, 56. 10.1038/s41392-021-00479-6 33563891 PMC7873034

[B259] Wu Q. J. Zhang T. N. Chen H. H. Yu X. F. Lv J. L. Liu Y. Y. (2022). The sirtuin family in health and disease. Signal Transduct. Target Ther. 7, 402. 10.1038/s41392-022-01257-8 36581622 PMC9797940

[B260] Wu Y. Dong J. H. Dai Y. F. Zhu M. Z. Wang M. Y. Zhang Y. (2023). Hepatic soluble epoxide hydrolase activity regulates cerebral Abeta metabolism and the pathogenesis of Alzheimer's disease in mice. Neuron 111, 2847–2862 e2810. 10.1016/j.neuron.2023.06.002 37402372

[B261] Xie J. Chen Y. Hu C. Pan Q. Wang B. Li X. (2017). Premature senescence of cardiac fibroblasts and atrial fibrosis in patients with atrial fibrillation. Oncotarget 8, 57981–57990. 10.18632/oncotarget.19853 28938531 PMC5601627

[B262] Yamamoto Y. Minami M. Yoshida K. Nagata M. Miyata T. Yang T. (2021). Irradiation accelerates plaque formation and cellular senescence in flow-altered carotid arteries of apolipoprotein E knock-out mice. J. Am. Heart Assoc. 10, e020712. 10.1161/JAHA.120.020712 34227406 PMC8483483

[B263] Yang H. H. Duan J. X. Liu S. K. Xiong J. B. Guan X. X. Zhong W. J. (2020). A COX-2/sEH dual inhibitor PTUPB alleviates lipopolysaccharide-induced acute lung injury in mice by inhibiting NLRP3 inflammasome activation. Theranostics 10, 4749–4761. 10.7150/thno.43108 32308747 PMC7163435

[B264] Yokoyama M. Shimizu I. Nagasawa A. Yoshida Y. Katsuumi G. Wakasugi T. (2019). p53 plays a crucial role in endothelial dysfunction associated with hyperglycemia and ischemia. J. Mol. Cell Cardiol. 129, 105–117. 10.1016/j.yjmcc.2019.02.010 30790589

[B265] Yoon Y. S. Yoon D. S. Lim I. K. Yoon S. H. Chung H. Y. Rojo M. (2006). Formation of elongated giant mitochondria in DFO-induced cellular senescence: involvement of enhanced fusion process through modulation of Fis1. J. Cell Physiol. 209, 468–480. 10.1002/jcp.20753 16883569

[B266] Yosef R. Pilpel N. Papismadov N. Gal H. Ovadya Y. Vadai E. (2017). p21 maintains senescent cell viability under persistent DNA damage response by restraining JNK and caspase signaling. EMBO J. 36, 2280–2295. 10.15252/embj.201695553 28607003 PMC5538795

[B267] Yosef R. Pilpel N. Tokarsky-Amiel R. Biran A. Ovadya Y. Cohen S. (2016). Directed elimination of senescent cells by inhibition of BCL-W and BCL-XL. Nat. Commun. 7, 11190. 10.1038/ncomms11190 27048913 PMC4823827

[B268] Yoshino J. Mills K. F. Yoon M. J. Imai S. (2011). Nicotinamide mononucleotide, a key NAD(+) intermediate, treats the pathophysiology of diet- and age-induced diabetes in mice. Cell Metab. 14, 528–536. 10.1016/j.cmet.2011.08.014 21982712 PMC3204926

[B269] Youle R. J. van der Bliek A. M. (2012). Mitochondrial fission, fusion, and stress. Science 337, 1062–1065. 10.1126/science.1219855 22936770 PMC4762028

[B270] Young A. R. Narita M. (2009). SASP reflects senescence. EMBO Rep. 10, 228–230. 10.1038/embor.2009.22 19218920 PMC2658552

[B271] Yousef A. Sosnowski D. K. Fang L. Legaspi R. J. Korodimas J. Lee A. (2024). Cardioprotective response and senescence in aged sEH null female mice exposed to LPS. Am. J. Physiol. Heart Circ. Physiol. 326, H1366–H1385. 10.1152/ajpheart.00706.2023 38578240

[B272] Yu W. Dittenhafer-Reed K. E. Denu J. M. (2012). SIRT3 protein deacetylates isocitrate dehydrogenase 2 (IDH2) and regulates mitochondrial redox status. J. Biol. Chem. 287, 14078–14086. 10.1074/jbc.M112.355206 22416140 PMC3340192

[B273] Zeng Z. Liang J. Wu L. Zhang H. Lv J. Chen N. (2020). Exercise-induced autophagy suppresses sarcopenia through akt/mTOR and akt/FoxO3a signal pathways and AMPK-mediated mitochondrial quality control. Front. Physiol. 11, 583478. 10.3389/fphys.2020.583478 33224037 PMC7667253

[B274] Zhang B. Cui S. Bai X. Zhuo L. Sun X. Hong Q. (2013). SIRT3 overexpression antagonizes high glucose accelerated cellular senescence in human diploid fibroblasts via the SIRT3-FOXO1 signaling pathway. Age (Dordr) 35, 2237–2253. 10.1007/s11357-013-9520-4 23494737 PMC3825003

[B275] Zhang C. Y. Duan J. X. Yang H. H. Sun C. C. Zhong W. J. Tao J. H. (2020). COX-2/sEH dual inhibitor PTUPB alleviates bleomycin-induced pulmonary fibrosis in mice via inhibiting senescence. FEBS J. 287, 1666–1680. 10.1111/febs.15105 31646730 PMC7174142

[B276] Zhang C. Y. Tan X. H. Yang H. H. Jin L. Hong J. R. Zhou Y. (2022). COX-2/sEH dual inhibitor alleviates hepatocyte senescence in NAFLD mice by restoring autophagy through Sirt1/PI3K/AKT/mTOR. Int. J. Mol. Sci. 23, 8267. 10.3390/ijms23158267 35897843 PMC9332821

[B277] Zhang C. Y. Zhong W. J. Liu Y. B. Duan J. X. Jiang N. Yang H. H. (2023). EETs alleviate alveolar epithelial cell senescence by inhibiting endoplasmic reticulum stress through the Trim25/Keap1/Nrf2 axis. Redox Biol. 63, 102765. 10.1016/j.redox.2023.102765 37269686 PMC10249012

[B278] Zhang F. X. Chen M. L. Shan Q. J. Zou J. G. Chen C. Yang B. (2007). Hypoxia reoxygenation induces premature senescence in neonatal SD rat cardiomyocytes. Acta Pharmacol. Sin. 28, 44–51. 10.1111/j.1745-7254.2007.00488.x 17184581

[B279] Zhang H. Ryu D. Wu Y. Gariani K. Wang X. Luan P. (2016). NAD⁺ repletion improves mitochondrial and stem cell function and enhances life span in mice. Science 352, 1436–1443. 10.1126/science.aaf2693 27127236

[B280] Zhang L. Elkahal J. Wang T. Rimmer R. Genzelinakh A. Bassat E. (2024). Egr1 regulates regenerative senescence and cardiac repair. Nat. Cardiovasc Res. 3, 915–932. 10.1038/s44161-024-00493-1 39196027

[B281] Zhang Y. El-Sikhry H. Chaudhary K. R. Batchu S. N. Shayeganpour A. Jukar T. O. (2009). Overexpression of CYP2J2 provides protection against doxorubicin-induced cardiotoxicity. Am. J. Physiol. Heart Circ. Physiol. 297, H37–H46. 10.1152/ajpheart.00983.2008 19429816 PMC2711738

[B282] Zhao J. He X. Zuo M. Li X. Sun Z. (2021). Anagliptin prevented interleukin 1β (IL-1β)-induced cellular senescence in vascular smooth muscle cells through increasing the expression of sirtuin1 (SIRT1). Bioengineered 12, 3968–3977. 10.1080/21655979.2021.1948289 34288819 PMC8806542

[B283] Zheng Z. Chen H. Li J. Li T. Zheng B. Zheng Y. (2012). Sirtuin 1-mediated cellular metabolic memory of high glucose via the LKB1/AMPK/ROS pathway and therapeutic effects of metformin. Diabetes 61, 217–228. 10.2337/db11-0416 22124463 PMC3237662

[B284] Zhu F. Li Y. Zhang J. Piao C. Liu T. Li H. H. (2013). Senescent cardiac fibroblast is critical for cardiac fibrosis after myocardial infarction. PLoS One 8, e74535. 10.1371/journal.pone.0074535 24040275 PMC3770549

[B285] Zhu M.-J. Wang X. Shi L. Liang L.-Y. Wang Y. (2018). Senescence, oxidative stress and mitochondria dysfunction. Med. Res. Innov. 21, 24. 10.15761/MRI.1000149

[B286] Ziegler D. V. Wiley C. D. Velarde M. C. (2015). Mitochondrial effectors of cellular senescence: beyond the free radical theory of aging. Aging Cell 14, 1–7. 10.1111/acel.12287 25399755 PMC4310776

[B287] Zimmermann A. Madreiter-Sokolowski C. Stryeck S. Abdellatif M. (2021). Targeting the mitochondria-proteostasis Axis to delay aging. Front. Cell Dev. Biol. 9, 656201. 10.3389/fcell.2021.656201 33777963 PMC7991595

